# NETosis inhibitory activity of LBW newborns’ cord blood plasma reverses lupus-associated immunopathology

**DOI:** 10.3389/fimmu.2026.1863546

**Published:** 2026-08-18

**Authors:** Hiral Thacker, Khushbu Priya, Uma Pandey, Shambhavi Singh, Manaswi Chaubey, Geeta Rai

**Affiliations:** 1 Department of Molecular and Human Genetics, Institute of Science, Banaras Hindu University, Varanasi, Uttar Pradesh, India; 2 Laboratory of Immunology, Molecular Immunology Section, National Eye Institute, National Institutes of Health, Bethesda, MD, United States; 3 Department of Obstetrics and Gynecology, Institute of Medical Sciences, Banaras Hindu University, Varanasi, Uttar Pradesh, India; 4 School of Medicine, D Y Patil University, Navi Mumbai, India; 5 Department of General Medicine, Institute of Medical Sciences, Banaras Hindu University, Varanasi, Uttar Pradesh, India

**Keywords:** inflammation, lupus mouse, NETosis, neutrophil extracellular trap, plasma, reactive oxygen species, systemic lupus erythematosus

## Abstract

**Background:**

Pathological NETosis contributes to immune dysregulation and organ damage in systemic lupus erythematosus (SLE), positioning it as a potential therapeutic target. Previous studies have shown suppressed NETosis in term low birth weight (tLBW) newborns. Several NETosis-suppressive molecules, including the α_1_-antitrypsin-derived C-terminal peptide and CRISPP have been reported in cord blood (CB) plasma and have shown suppressive effects on NET formation. Given the pathogenic role of excessive NETosis in SLE, these observations suggest that LBW newborns’ CB plasma (LP) may contain putative NETosis-inhibitory factors with therapeutic potential and support further investigations.

**Objectives:**

To evaluate the NETosis-inhibitory activity of LP and its effects on lupus-associated pathological features in neutrophils from patients with SLE and in the pristane-induced lupus (PIL) mouse model.

**Methods:**

*Ex vivo* effects of LP on NETosis were evaluated using neutrophils from patients with SLE (n=35) and PIL mouse (n=18; n=6/group). NET formation was assessed by extracellular DNA release and expression of key markers, including citrullinated histone H3 (CitH3), neutrophil elastase (NE), and myeloperoxidase (MPO), along with cytoplasmic and mitochondrial ROS production. *In vivo* efficacy was determined by measuring reduction in NETosis, oxidative stress, anti-dsDNA autoantibody levels, proteinuria, and improvement of tissue oxygenation and vascularization in the heart, liver and kidneys of PIL mice.

**Results:**

LP significantly reduced NET formation, as evidenced by reduced extracellular DNA release and expression of CitH3 and NE in SLE neutrophils. Extracellular DNA release decreased by 13% and 16.66% along with a reduction in netting neutrophils by 27.4% and 21.12% under basal and LPS-stimulated conditions, respectively. LP also suppressed ROS production in SLE neutrophils. In PIL mice, LP reduced NETosis (30.93%) and oxidative stress (15.34%mitochondrial ROS; 24.28% cytoplasmic ROS) in blood-derived neutrophils, decreased MPO and Sytox Orange signal intensities in the heart, liver, and kidneys. LP further decreased anti-dsDNA autoantibody levels (57.9%) and proteinuria (93.59%), preserved vascular integrity, and improved tissue oxygenation.

**Conclusion:**

LP exhibits NETosis-inhibitory activity and ameliorates lupus-associated pathology in the PIL mouse and *ex vivo* SLE neutrophil system. These findings suggest the presence of as-yet-uncharacterized endogenous plasma components that modulate NET formation and support further investigation as potential therapeutics for SLE.

## 1 Introduction

The immune system relies on a tightly regulated balance between protective host defense and controlled resolution of inflammation; disruption of this equilibrium can lead to pathological immune activation and autoimmunity. Systemic lupus erythematosus (SLE) exemplifies this complex imbalance, arising from dysregulated crosstalk between innate and adaptive immune responses, which drives chronic inflammation and multi-organ damage. Affecting over 3.4 million individuals worldwide and disproportionately impacting women of reproductive age, SLE remains a significant global health burden (1, 2). Despite the use of conventional immunotherapies, including corticosteroids and immunosuppressive agents, that effectively control disease activity, their long-term use is constrained by significant adverse effects and fails to address the underlying pathogenic mechanisms adequately. In this context, neutrophil dysfunction has emerged as an important contributor to SLE pathogenesis, extending beyond its traditional role in host defense (3). In particular, NETosis, a distinct form of programmed neutrophil death, has been reported to be an important contributor to disease progression (4, 5). Excessive or dysregulated NET formation, coupled with impaired clearance, leads to the accumulation of immunogenic nuclear components that drive autoantibody production, immune complex deposition, and amplification of inflammatory signaling pathways (6, 7). Beyond their antigenic role, NETs actively amplify inflammation by stimulating plasmacytoid dendritic cells, enhancing type I interferon responses, and activating autoreactive B and T cells, thereby reinforcing the autoimmune cascade (8). Moreover, NETs contribute to endothelial dysfunction, thrombosis, and tissue injury, linking innate immune dysregulation to organ damage, including lupus nephritis and cardiovascular complications (9, 10). Given the pivotal role of oxidative signaling in NET formation, excessive reactive oxygen species (ROS) production not only drives NETosis but also exacerbates tissue damage and immune activation, thereby sustaining disease progression. These insights highlight NETosis and associated oxidative stress pathways as important contributors to lupus pathogenesis and underscore the importance of investigating approaches capable of modulating NET-driven inflammation. Given the central role of neutrophils in inflammatory pathology, context-dependent alterations in their function, ranging from aberrant activation to impaired responses, play a critical role in shaping immune function during early life, particularly in vulnerable neonatal populations. The neonatal immune system is exposed to extensive antigenic stimuli during the early postnatal period (11). Rapid environmental changes and microbial colonization during this period critically shape the neonatal immune response. According to the World Health Organization, low birth weight (LBW) is defined as a birth weight of less than 2500 g. Globally, more than 20 million LBW newborns are born each year (12), with a mortality risk approximately 20-fold higher than that of normal birth weight (NBW) newborns. Survivors frequently experience severe complications, including jaundice, respiratory distress, immunodeficiency, and sudden infant death syndrome (SIDS).

Our previous studies have demonstrated that term LBW (tLBW) newborns exhibit compromised innate immunity, characterized by severely impaired NETosis (13). Transcriptomic profiling has revealed significant downregulation of genes involved in neutrophil effector functions in tLBW newborns (14, 15). Furthermore, we identified attenuated GPCR-mediated signaling pathways, including C5aR1 and LTB4R1, along with reduced expression of NET-associated proteins, collectively impairing NET formation in newborns with LBW (16). However, the mechanisms underlying impaired NETosis in tLBW newborns remain incompletely understood. Reduced NET formation may arise from intrinsic developmental characteristics of neonatal neutrophils, soluble circulating mediators, or a combination of both. Although impaired NETosis in tLBW newborns has largely been attributed to intrinsic alterations in neutrophil signaling pathways, the contribution of soluble plasma-derived regulatory mechanisms remains unknown.

**Figure 1:**
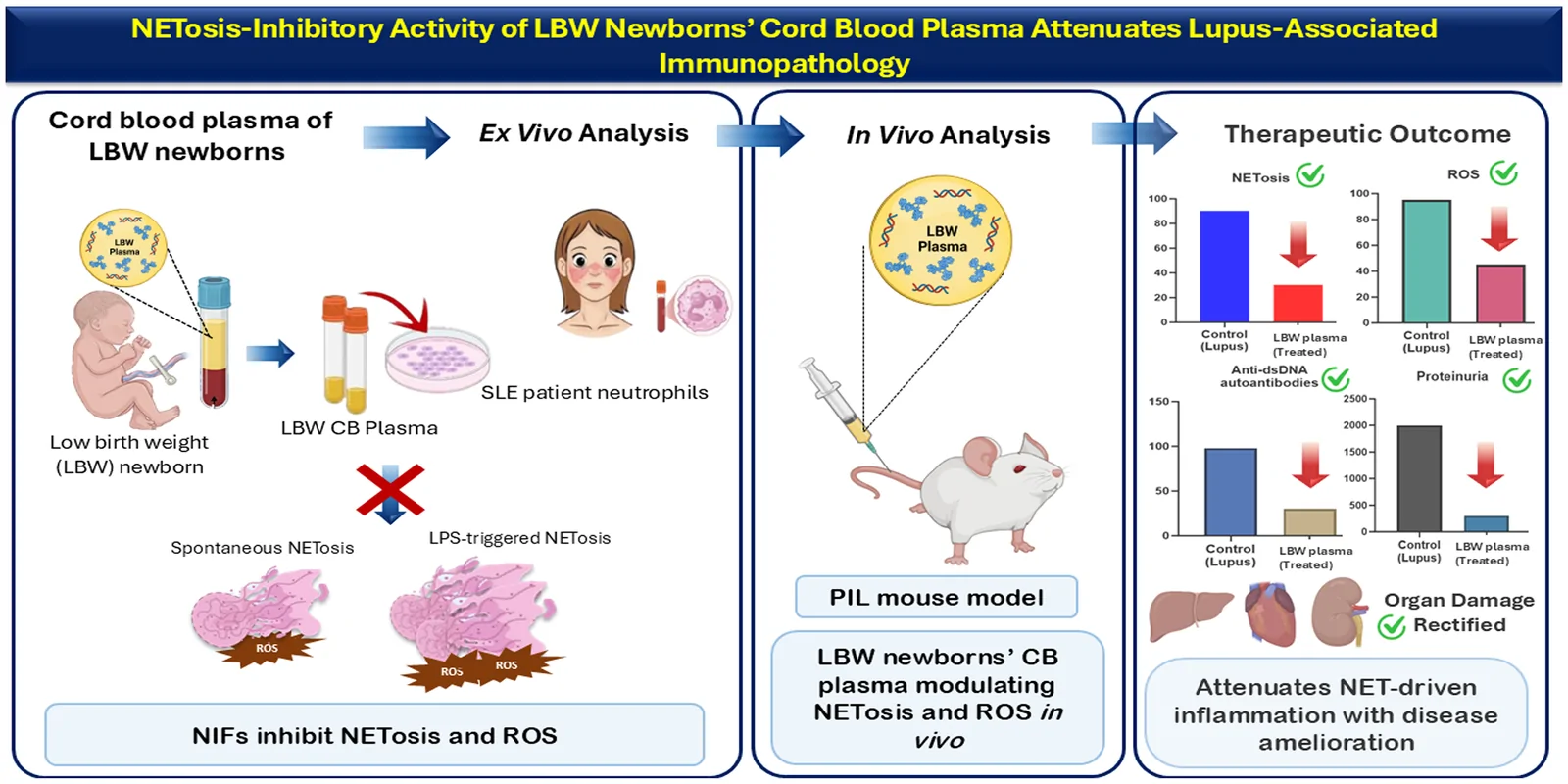
Graphical abstract illustrating the experimental workflow for evaluating the NETosis-inhibitory activity of LP in modulating NETosis in neutrophils from SLE patients and *in vivo* assessment in the PIL mouse model. This schematic depicts analyses of NETosis, inflammatory responses, serum anti-dsDNA autoantibody levels, proteinuria, and disease-associated phenotypes.

Importantly, neonatal NET-inhibitory factors (nNIFs) have previously been identified in cord blood and shown to transiently suppress NET formation during early life, thereby limiting excessive inflammatory responses (17). These observations raise the possibility that cord blood plasma from tLBW newborns may contain soluble NET-inhibitory factors capable of modulating pathological NETosis. However, whether such inhibitory activity exists in tLBW cord blood plasma and whether it can influence NET-driven autoimmune pathology has not been explored. In the present study, we investigated the NETosis-inhibitory activity of LP and its ability to suppress aberrant NETosis in neutrophils from patients with SLE and PIL mouse model. We show that LP significantly attenuates NET formation, as evidenced by reduced extracellular DNA release and decreased expression of key NET-associated markers, including citrullinated histone H3 (CitH3), neutrophil elastase (NE), and myeloperoxidase (MPO), along with suppression of cytoplasmic and mitochondrial ROS production. Furthermore, the therapeutic relevance of these findings was validated *in vivo* using a pristane-induced lupus (PIL) mouse model, where administration of LP led to a significant suppression of NETosis and oxidative stress across the heart, liver, and kidneys. The observed NET attenuation was accompanied by a reduction in autoantibody levels, decreased proteinuria, preservation of vascular integrity, and reduced tissue injury. The graphical abstract illustrating the workflow for evaluating the NETosis - inhibitory activity of LP in modulating NETosis is depicted in **Figure 1**. These observations suggest the potential NETosis inhibitory activity of LPand support further investigation into the molecular factors responsible for these effects and their potential relevance in NET-driven autoimmune diseases.

## 2 Materials and methods

### 2.1 Study design, subjects, and ethical statement

*Ex vivo* NET assessment studies were performed by recruiting patients with SLE (n = 35) and term newborns (n = 40; 20 NBW and 20 LBW) from the Sir Sunder Lal Hospital, Banaras Hindu University (BHU; Varanasi, India). Healthy adults (n = 10) were included as controls. Ethical clearance was obtained from the Institutional Ethics Committee of the Institute of Science, BHU (Ref. No. I.Sc./ECM-XII/2021–22).

#### 2.1.1 Sample collection and clinical assessment

Fresh CB from term newborns (gestational age >37 weeks), peripheral blood from patients with SLE, and healthy adults were collected after obtaining written informed consent, in accordance with ethical guidelines. Detailed demographic and clinical information, including age, sex, medical history, symptoms, medications, and clinical manifestations, was recorded using a pre-designed proforma.

The inclusion criteria for this study were: age >18 years; confirmed diagnosis of SLE based on the 2019 EULAR/ACR classification (European Alliance of Associations for Rheumatology/American College of Rheumatology) criteria; and the presence of other clinical manifestations.

The primary exclusion criteria included patients with a history of other comorbid conditions, including hepatitis B or C, HIV, cancer, diabetes, or an HbA1c level >6%, as well as those with any significant medical or psychiatric comorbidities or receiving related treatments.

For umbilical CB collection, only pregnant women with term gestation and no infections or pregnancy-related complications—including diabetes, hypo- or hyperthyroidism, preeclampsia, or a history of hepatitis B or C, HIV, or HBsAg positivity during pregnancy—were enrolled.

#### 2.1.2 Umbilical CB plasma preparation

Immediately after delivery, umbilical CB was collected in a sterile EDTA tube and processed for plasma isolation. Briefly, 10 mL of blood was centrifuged at 1,500 × g for 10 min at 4 °C to separate the plasma. The supernatant was carefully transferred to a fresh tube without disturbing the buffy coat and subjected to a second centrifugation at 12,000 × g for 10 min to remove residual cellular debris. The clarified plasma was aliquoted and stored at −80 °C until use.

#### 2.1.3 Isolation of neutrophils

Neutrophils were isolated from the peripheral blood of patients with SLE and healthy controls using density-gradient centrifugation. Briefly, whole blood was layered over HiSep™ LSM 1077 (LS001; HiMedia, Mumbai, India) and GranuloSep™ GSM 1119 (LS004; HiMedia, Mumbai, India) and centrifuged at 700 × g for 30 min, according to the manufacturer’s instructions. The granulocyte-rich lower layer was collected, subjected to red blood cell (RBC) lysis, washed with PBS, and resuspended in Roswell Park Memorial Institute (RPMI)-1640 supplemented with 10% low-endotoxin fetal bovine serum (FBS) (10270106, Gibco, Waltham, USA). The mean purity (> 95%) of neutrophils was assessed by differential counting following Diff-Quik staining. Isolated neutrophils were immediately used for functional assays, which were initiated within 1 hour of sample collection.

#### 2.1.4 NET formation assay by CB plasma

Isolated neutrophils (1 × 10^6^ cells/mL) were pre-incubated with CB-derived plasma from both NBW and LBW newborns for 1 h before NET induction. The cells were then washed with PBS and cultured with or without LPS (1 µg/mL) (L2630, Sigma-Aldrich, St. Louis, USA) on poly-L-lysine (P4707, Sigma-Aldrich, St. Louis, USA) coated glass coverslips for 3 h. After NET induction, neutrophils were washed and stained for extracellular DNA release using SYTOX Orange (red) dye (S11368, Molecular Probes, Eugene, USA) at a final concentration of 0.5 µM. SYTO 13 (green) (S7575, Molecular Probes, Eugene, USA) was used to stain the intracellular DNA. A laser-scanning super-resolution confocal microscope (Leica, Wetzlar, Germany) was used to acquire images. Image processing was performed using ImageJ software.

#### 2.1.5 Qualitative assessment of NETs by fluorescence microscopy

For NET visualization, neutrophils (1 × 10^6^ cells/mL) from patients with SLE were seeded onto 0.001% poly L-lysine-coated glass coverslips after preincubation with CB plasma from LBW and NBW newborns and were left uninduced or induced with LPS for 3 h. Cells were fixed with 4% paraformaldehyde for 30 min after NET induction and rinsed with phosphate-buffered saline (PBS). The cells were permeabilized with 0.1% Triton X-100 for 5 min and blocked for 1 h with 5% BSA in PBST. Next, the cells were incubated overnight with two primary antibodies – rabbit anti-human CitH3 [1:250] (Abcam) and mouse anti-human NE [1:100] (Santa Cruz Biotechnology, Santa Cruz, CA) in PBST containing 5% BSA. The next day, after washing with PBST, the cells were incubated with FITC-conjugated goat anti-rabbit IgG secondary antibody [1:300] (Santa Cruz Biotechnology, Santa Cruz, CA) for anti-CitH3 and TRITC-conjugated goat anti-mouse IgG secondary antibody [1:500] (GeNei™, Bengaluru, Karnataka) for anti-NE. Finally, the cells were stained with DAPI (1 µg/mL) (Sigma-Aldrich, St. Louis, MO) for 20 min, washed with PBS, and mounted with DABCO (Sigma-Aldrich, St. Louis, MO). The slides were visualized using a laser-scanning super-resolution confocal microscope (Leica, Wetzlar, Germany). Image processing was performed using ImageJ software.

#### 2.1.6 Extracellular DNA assay

Plasma-incubated, uninduced, and induced neutrophils (1 × 10^6^ cells/mL) were seeded in a 96-well black plate and cultured for 3 h. To analyze NETs and extracellular DNA release, 100 µL of supernatant from each well was incubated with SYTOX Orange (0.5 µM), and fluorescence was measured using a multimode microplate reader (Synergy™ Mx, BioTek, Vermont, USA) at 547/570 nm using Gen5 2.01.14 software.

#### 2.1.7 Flow cytometry assay to detect NETs

To detect NETs using flow cytometry, neutrophils (1 × 10^6^ cells/mL) with or without LPS induction following plasma incubation were fixed with 2% paraformaldehyde, washed, and permeabilized with FACS permeabilizing solution (BD Biosciences) for 10 min on ice. Blocking was performed with 2% BSA in PBS, and the cells were incubated with PE-conjugated anti-human MPO antibody (IM3455U, Beckman Coulter, California, USA) according to the manufacturer’s guidelines at 4 °C for 30 min in the dark. After washing, the cells were resuspended in 2% BSA in PBS containing 100 ng/mL DAPI and incubated for 20 min at 4 °C. Following centrifugation, the cells were resuspended in PBS and immediately analyzed using a CytoFLEX LX flow cytometer (Beckman Coulter). Data analysis was performed using CytExpert software (Beckman Coulter).

#### 2.1.8 Evaluation of cytotoxicity

To evaluate whether CB plasma has any cytotoxic effect on neutrophils, an MTT (3-[4,5-dimethylthiazol-2-yl]-2,5-diphenyl tetrazolium bromide) assay was performed. Briefly, plasma-incubated neutrophils (1 × 10^6^ cells/mL) were treated with MTT (5 mg/mL) for 4 h. Cellular metabolic activity was assessed by dissolving formazan crystals in 0.04 M HCl in isopropanol. Absorbance was quantified at 570 nm using a multimode microplate reader (Synergy™ Mx, BioTek, Vermont, USA) with the Gen5 2.01.14 software.

### 2.2 Animals

Female BALB/c mice (n = 24; 8 weeks old; weighing 20 ± 2 g) were procured from the Institute of Medical Sciences, BHU (Varanasi, India). The mice were housed under specific pathogen-free conditions with an alternating 12-hour light/dark cycle. Relative humidity was maintained at 50 ± 5%, and the temperature was kept constant at 25 °C. All treatment groups were age-matched, and experimental protocols involving the care and use of animals were conducted in compliance with the Institutional Animal Ethical Committee (IAEC) guidelines (Ref no. BHU/DoZ/IAEC/2022-23/003). 

#### 2.2.1 Development of the PIL model

The PIL model was selected because it recapitulates several clinical features of human SLE, including autoantibody production, proteinuria, systemic inflammation, and enhanced NETosis. To establish the lupus mouse model, mice were randomly allocated to pristane (n=18) or control (n = 6) groups. Lupus-like disease was induced by a single intraperitoneal injection of pristane (0.5 mL; Sigma-Aldrich). Animals were monitored longitudinally, and after 4 weeks, PIL mice were systematically evaluated for disease development, including autoantibody production, proteinuria, body weight changes, and limb inflammation. Comprehensive physiological assessments, encompassing organ pathology, oxygen saturation, and vascular alterations, were also performed to confirm lupus-like pathology.

Four weeks after pristane administration, disease establishment was confirmed through assessment of proteinuria, autoantibody production, limb inflammation, and physiological alterations. Treatment was initiated immediately after disease confirmation.

Upon validation of disease establishment, PIL mice were randomly assigned treatment groups (n = 6 per group): (i) saline-treated PIL controls, (ii) PIL mice administered low birth weight (LBW) newborns’ CB plasma, and (iii) PIL mice administered normal birth weight (NBW) newborns’ CB plasma. Plasma from NBW newborns was included as a biological control to determine whether any observed NETosis-inhibitory activity was specific to LP rather than a general property of neonatal cord blood plasma. To minimize donor-dependent variability, plasma samples were pooled before use.

**Figure 2:**
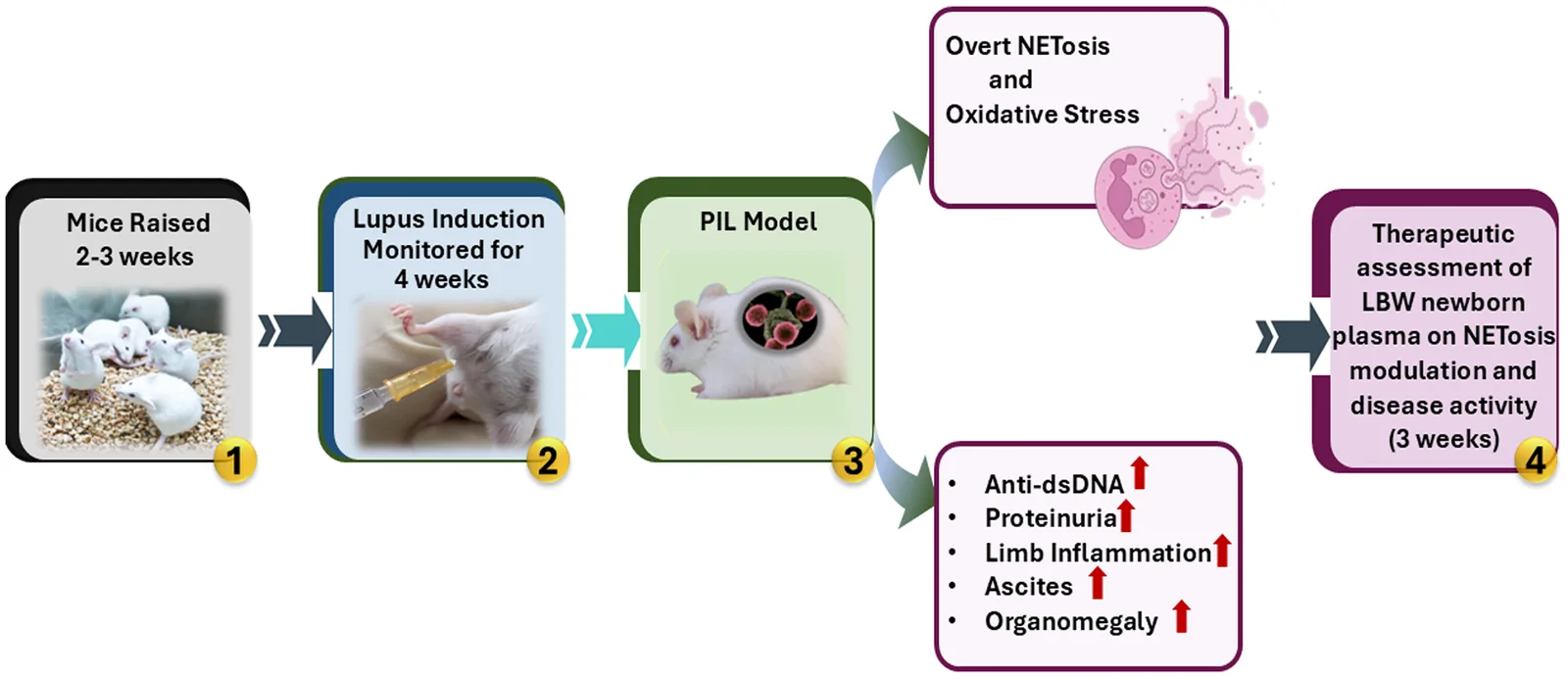
Experimental layout for the preclinical evaluation of LBW newborns’ cord blood plasma in a pristane-induced lupus (PIL) mouse model, modulating NETosis and lupus-associated pathology.

Mice received 150 µL of pooled plasma via intravenous injection on alternate days for 3 weeks. Age-matched healthy mice (n = 6) served as non-diseased controls and received normal saline under identical conditions. The experimental layout is described in **Figure 2**.

#### 2.2.2 Clinical validation of the PIL model

##### 2.2.2.1 Determination of autoantibodies

Four weeks following the injection of pristane, autoantibodies targeting nuclear antigens were identified in the serum of PIL mice. Retro-orbital blood samples were used to measure serum IgG levels against dsDNA, utilizing ELISA quantification kits (MyBioSource) in accordance with the manufacturer’s guidelines.

##### 2.2.2.2 Proteinuria and body weight

To evaluate proteinuria, urine samples were collected from each mouse, including the control group, using clean microcentrifuge tubes. The samples were then centrifuged at high speed to eliminate any sediment. The resulting clear urine supernatant was tested with a urine analysis strip (Uristix, Siemens) according to the manufacturer’s guidelines. Additionally, the body weight of all mice was accurately recorded both before and after the induction of the disease.

##### 2.2.2.3 Limb inflammation and ascites development

After administering pristane via intraperitoneal injection, the mice were evaluated for paw swelling and inflammation without knowledge of their identities; redness in both the hind and forelimbs served as an indicator of inflammation severity. Additionally, these mice were observed for the onset of ascites in the peritoneal cavity, a hallmark of the SLE mouse model. Two other specialists independently assessed all clinical signs, with the mice’s identities kept hidden.

##### 2.2.2.4 NETosis and ROS

To mirror the pathological features of SLE, particularly sustained NETosis and elevated ROS generation, the lupus mouse model was systematically assessed for these hallmarks. After one month of post-pristane administration, neutrophils isolated from retro-orbital blood were analyzed for spontaneous NET formation and associated inflammatory responses. Concurrently, oxidative stress levels were measured and compared with those of healthy controls to confirm enhanced ROS production in diseased mice.

#### 2.2.3 Mouse neutrophil purification

After disease induction and completion of the treatment period (3 weeks), purified circulating neutrophils were isolated from retro-orbital blood by carefully inserting a sterile capillary tube into the retro-orbital sinus of the mice. After dilution with an equal volume of PBS, blood samples were subjected to a discontinuous ficoll gradient (1.077 and 1.119 g/mL). Neutrophils were collected from the interface and subjected to RBC lysis to remove red blood cells. The neutrophil purity and viability were greater than 95%.

For assessment of the NETosis-inhibitory role of plasma, mice were euthanized at 3 weeks post-treatment by CO_2_ inhalation using a gradual-fill method followed by cervical dislocation as a secondary method to ensure death while minimizing pain and distress. Organs were then harvested. Mice were placed in the supine position, and vital organs, including the heart, lungs, spleen, liver, and kidneys, were carefully dissected and collected in clean, labeled Petri dishes. Organ length (cm) was measured using a ruler by recording the maximum dimension from one end to the other. To preserve cellular integrity, organs were immediately immersed in 10% formalin and maintained until further processing.

#### 2.2.4 Quantification of ROS

Cellular ROS and mitochondrial ROS production were detected using dichlorodihydrofluorescein diacetate (DCFDA) (D6883, Sigma-Aldrich, St. Louis, USA) and MitoSOX Red (M36008, Molecular Probes, Eugene, USA), respectively. MitoSOX Red is a fluorogenic dye specifically targeted to mitochondria in live cells. DCFDA is a widely used fluorogenic dye that facilitates cell permeability, allowing for improved loading and trapping. Upon oxidation, it emits green fluorescence as an indicator of cellular ROS production. Briefly, plasma-incubated uninduced and LPS-induced neutrophils (1 × 10^6^ cells/mL) were cultured in a 96-well plate. Mitochondrial and cellular ROS were detected by incubating cells with MitoSOX Red and DCFDA, respectively, and quantified using a multimode microplate reader (Synergy™ Mx, BioTek, Vermont, USA) with the Gen5 2.01.14 software.

#### 2.2.5 *In vivo* multispectral Photoacoustic Imaging to quantify NETs

*In vivo* NETosis was assessed using photoacoustic imaging (PAI), a noninvasive tool that provides excellent optical contrast with a spatial resolution similar to that of ultrasound. The Vevo LAZR-X system was applied to a phantom to evaluate the effectiveness of photoacoustic contrast agents, including SYTOX Orange and anti-MPO antibody, in soft tissue–mimicking phantom images. To determine whether these agents could be used *in vivo* as optoacoustic agents, their absorption spectra were analyzed using the spectral mode of the system.

A Vevo3100-LAZRX small animal PAI system with a 250S transducer (15–30 MHz, scan depth of 30 mm) for the cardiac region and an MX-550D linear-array transducer (25–50 MHz, scan depth of 15 mm) for the abdominal region was used to perform high-resolution ultrasound imaging. The imaging parameters were maintained at high sensitivity, with a step size of 0.279 mm, laser power set to 100%, and photoacoustic gain of 37 dB. Abdominal hair was removed using a hair removal cream, and alcohol swabs were used to clean the superficial dermal layer. A clear, bubble-free ultrasonic gel (OXD) was used to fill a 5 mm space between the mouse skin and transducer surface. During imaging, the mice were anesthetized using 2% isoflurane for induction and 1.5–2% for maintenance. For ultrasound and photoacoustic imaging, the mice were placed in a supine position on an operating table maintained at 37 °C. Physiological parameters, including heart rate, respiratory rate, and body temperature, were monitored. B-mode imaging, which provides a real-time cross-sectional view of internal organs, including the kidneys, liver, and heart, was used to visualize organ structure.

Polymeric blood vessel–mimicking tubes were used as optically diffusive phantoms, and their optical characteristics and fluorochrome biodistribution closely resembled those of living tissues. SYTOX Orange- or MPO-filled narrow tubes representing blood vessels were immersed in cylindrical phantom structures. For comparison, a blank tube and a tube filled with water were included. The evaluation of fluorescence signals and related wavelengths for SYTOX Orange and MPO was enabled by the optical properties of these phantoms. Normal saline was used to dilute SYTOX Orange and the targeted anti-MPO antibodies to concentrations of 1 nmol and 100 µg/mL, respectively. After injecting these agents into the blood vessel–mimicking tubes, PAI was performed in conjunction with standard ultrasound imaging. The strength of the PA signal was measured between 680 and 700 nm, and the optimal wavelength was subsequently used to investigate fluorochrome accumulation in the liver, kidneys, and heart of control, lupus, LBW, and NBW newborns’ CB plasma–treated mice.

To evaluate NET protein markers, PE-labeled anti-MPO antibody (Beckman Coulter) was administered for 30 min at 37 °C, and SYTOX Orange (1 nmol/mouse, i.v.) was administered to assess extracellular DNA release after 60 min of imaging. The images were recorded and merged to generate a single dual-color image.

#### 2.2.6 Statistical analysis

GraphPad Prism (version 10.1.2) was used to perform the statistical analyses. One-way and two-way ANOVAs were used to compare two or more groups, respectively. For all experiments, at least three independent replicates were performed, and the data are presented as the mean ± SD. Specific p-values are provided in the figure legends. Differences were considered statistically significant at p < 0.05.

## 3 Results

### 3.1 Subject demographics

CB plasma samples: CB samples were collected immediately after delivery from newborns (n = 40; 20 LBW and 20 NBW) with term gestation (≥ 37 weeks) following written informed consent. Details of the newborns, including birth weight, gestational age, maternal age, and mode of delivery, are summarized in the **Supplementary Table 1**.

Patients with SLE (n = 35) who fulfilled the American College of Rheumatology classification criteria and were registered at the Institute of Medical Sciences, BHU, were enrolled in this study. The **Supplementary Table 2** presents the clinical profiles of the included patients.

#### 3.1.1 LP suppressed NET formation and associated marker expression in uninduced and LPS-induced neutrophils from SLE patients’ *ex vivo*

**Figure 3:**
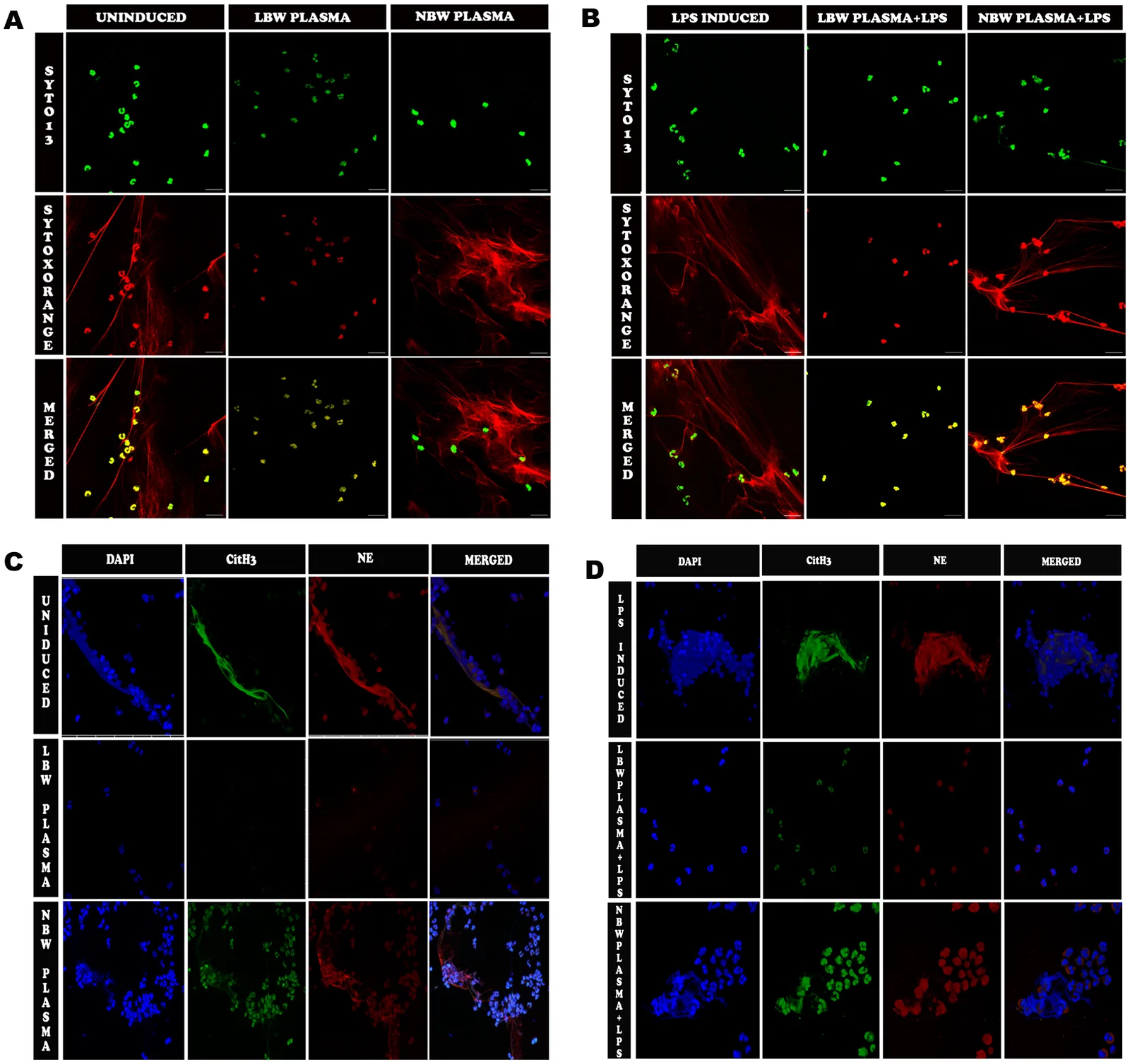
Evaluation of NETosis inhibition by LP in neutrophils from SLE patients. Representative data show the effects of LBW newborns’ cord blood plasma (LBW plasma) on NET formation and NETosis marker expression in unstimulated and LPS-stimulated neutrophils from SLE patients. **(A, B)** Confocal microscopy images depict NET formation assessed by extracellular DNA staining (SYTOX Orange) and a cell-permeant intracellular DNA-binding dye (Syto13) in unstimulated **(A)** and LPS-stimulated **(B)** neutrophils (63× magnification; scale bar = 20 µm; n = 3). **(C, D)** Immunofluorescence images show expression of NETosis markers CitH3 and NE in unstimulated **(C)** and LPS-stimulated **(D)** neutrophils after incubation with LBW plasma. Netting neutrophils were confirmed by co-localization of NE (red), CitH3 (green), and DNA (blue) (63× magnification; scale bar = 20 µm). Data are representative of three independent experiments.

Persistent NETosis in patients with SLE fuels inflammation and exacerbates disease progression. We found that CB plasma from LBW newborns suppressed the spontaneous extracellular DNA release from neutrophils of SLE patients (**Figure 3A**) or lipopolysaccharide (LPS)- induced (**Figure 3B**) conditions, whereas plasma from NBW newborns showed no inhibitory effect, highlighting the NET-inhibitory activity of LP in SLE.

We further examined the effect of LP on NETosis markers, CitH3 (green) and NE (red). Notably, the confocal microscopy images revealed that LP significantly reduced the expression of these NET-associated markers under both basal (**Figure 3C**) and LPS-stimulated (**Figure 3D**) conditions compared to NBW newborns’ CB plasma, further supporting its inhibitory effect on NETosis-associated inflammatory signatures.

#### 3.1.2 Quantitative measurements show suppression of NETosis and ROS generation in SLE patients’ neutrophils treated with LP

**Figure 4:**
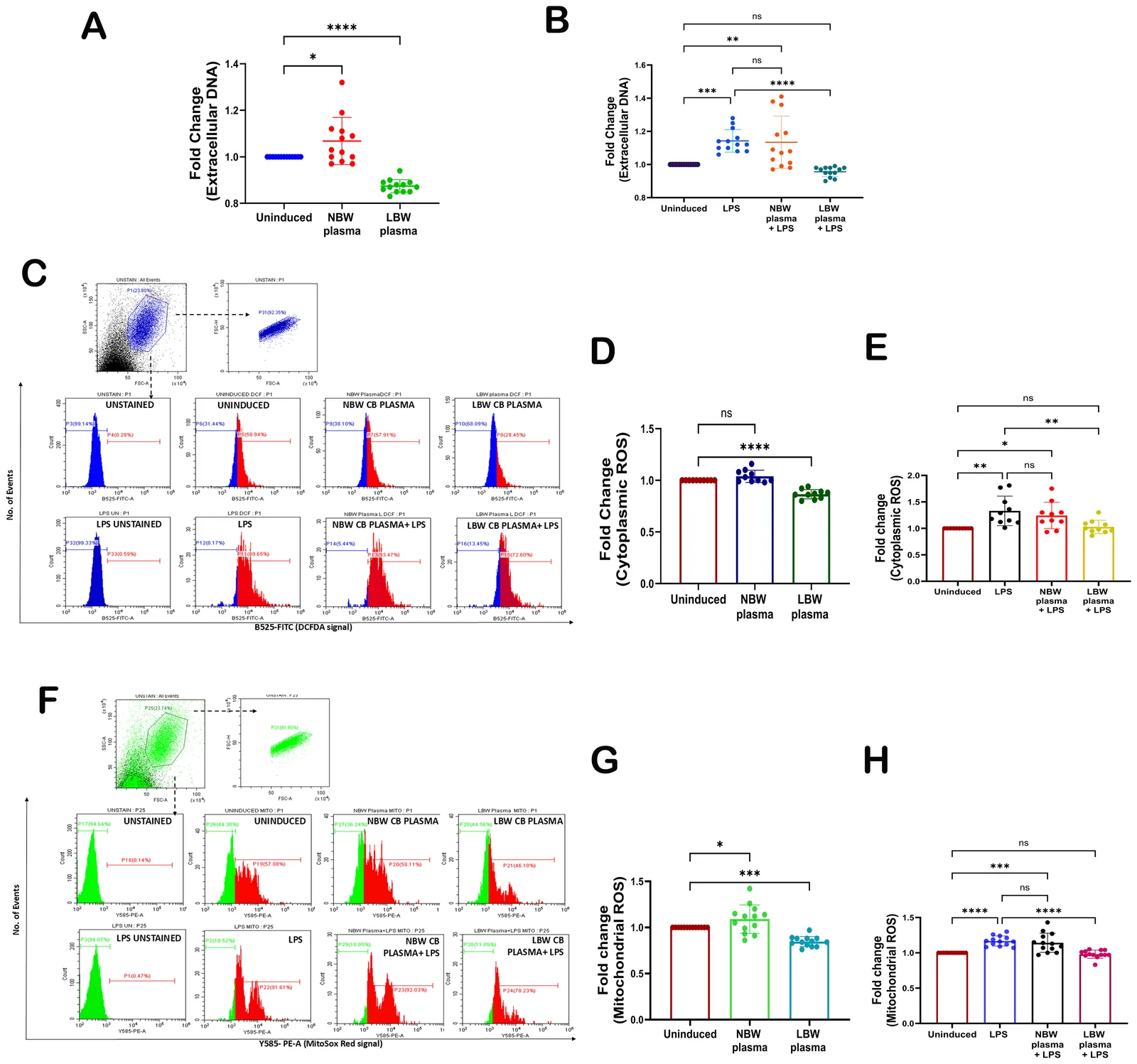
Quantification of extracellular DNA release and ROS production in neutrophils from SLE patients following incubation with LBW newborns’ CB plasma. Representative data showing extracellular DNA release in both unstimulated **(A)** and LPS-stimulated **(B)** neutrophils (n = 13). Cytoplasmic ROS [**(C–E)**; n = 10] and mitochondrial ROS [**(F–H)**; n = 13) levels in both unstimulated and LPS-stimulated neutrophils. For Flow cytometry histograms, the x-axis represents the fluorescence intensity of DCFDA and MitoSox Red, and the y-axis represents the number of events (Count). All data are presented as mean ± SD. Statistical analysis was performed using one-way ANOVA, with significance indicated as: non-significant (ns), p > 0.05, *p < 0.05, **p < 0.01, ***p <0.001, and ****p < 0.0001.

To complement our qualitative confocal microscopy observations, NETosis was quantitatively assessed by measuring extracellular DNA in cell culture supernatants using a multimode plate reader following treatment with LBW newborns’ CB plasma. Analysis revealed a 13% reduction under basal conditions (**Figure 4A**) and a 16.66% reduction following LPS stimulation in neutrophils from SLE patients (**Figure 4B**). These findings are in concurrence with the microscopy data, highlighting the inhibitory activity of LBW plasma capable of restraining aberrant neutrophil activation in SLE.

Given the close association between NET formation and ROS generation via NADPH oxidase (NOX)-dependent and mitochondrial pathways, we next examined the effect of LP on ROS production. Treatment of SLE neutrophils with LP under both basal and LPS-stimulated conditions resulted in a marked reduction in cytoplasmic (**Figures 4C–E**) and mitochondrial (**Figures 4F–H**) ROS levels. These findings indicate that LP not only limits NETosis but also mitigates ROS-driven oxidative stress, underscoring its immunomodulatory potential in SLE.

#### 3.1.3 LP reduced the percentage of netting neutrophils

**Figure 5:**
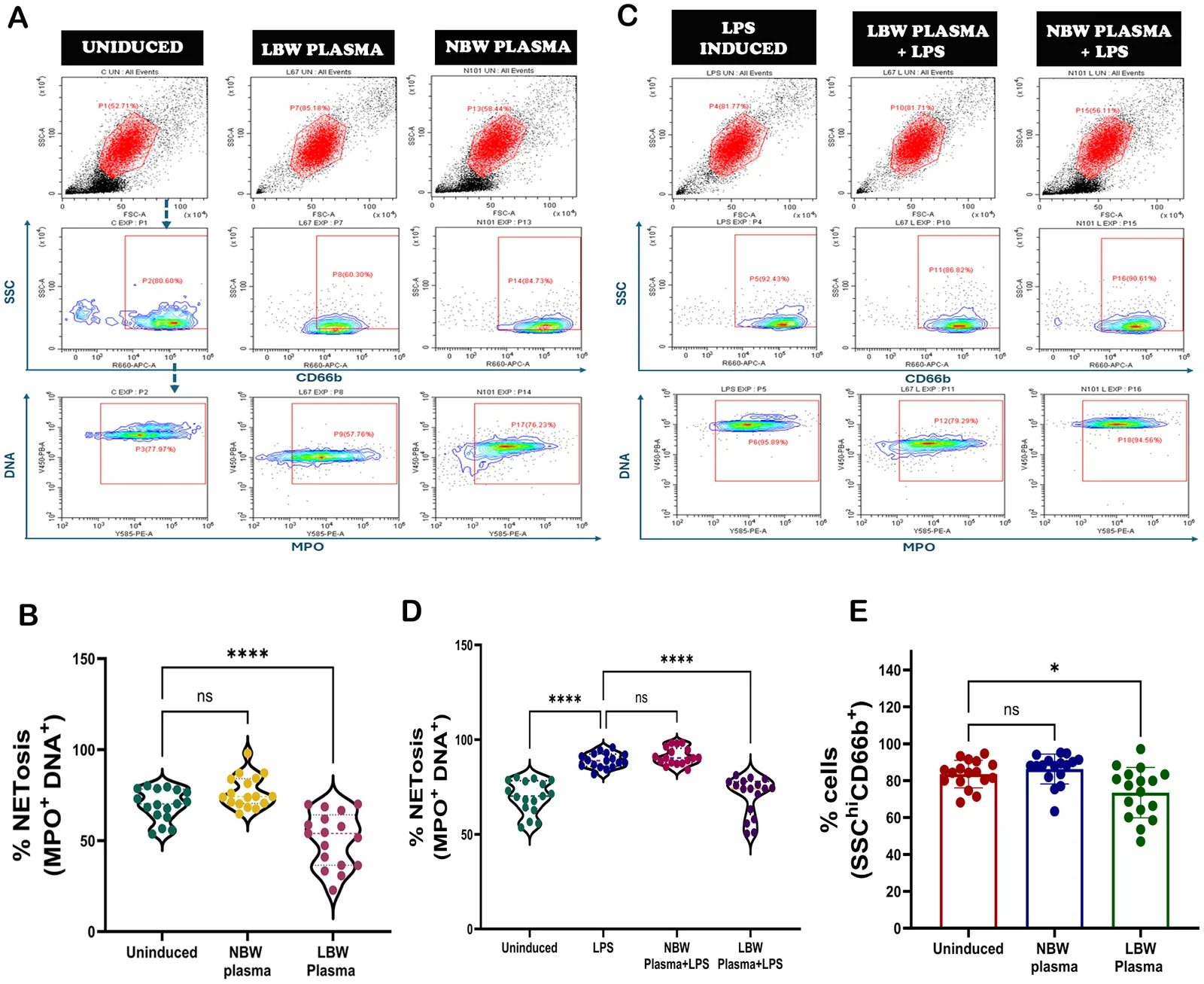
Flow cytometry-based assessment of the effect of LBW cord blood **(CB)** plasma on NETosis in neutrophils from SLE patients. Representative flow cytometry plots showing MPO–DNA-positive (netting) neutrophils in **(A, B)** unstimulated and **(C, D)** LPS-stimulated neutrophils from SLE patients following incubation with LP. **(E)** Quantification of neutrophil activation. Data are presented as violin plots and bar graphs (mean ± SD; n = 17). Statistical analysis was performed using one-way ANOVA, with significance indicated as: non-significant (ns), p > 0.05; *p < 0.05; **p < 0.01; ***p < 0.001; **p < 0.0001.

We evaluated the effect of LP on the propensity of neutrophils to undergo NET formation. A flow cytometry–based high-throughput assay was employed to quantify the percentage of netting neutrophils by detecting MPO–DNA complexes, which are a hallmark of NETosis. Neutrophils isolated from SLE patients were incubated with plasma from LBW and NBW newborns and subsequently assessed under both basal (unstimulated) and LPS-stimulated conditions. Notably, treatment with LP was associated with a significant reduction in the proportion of MPO–DNA–positive neutrophils, with NETosis reduced by 27.4% compared with the uninduced control (**Figures 5A, B**) and by 21.12% compared with LPS-stimulated neutrophils (**Figures 5C, D**). Along with this, reduced expression of CD66b was observed in neutrophils treated with LP, suggesting decreased activation (**Figure 5E**). These findings indicate that LP possesses NETosis-inhibitory activity by directly attenuating NET formation, thereby interrupting an important pathogenic driver of autoimmunity in SLE.

#### 3.1.4 LP dampens NETosis in LPS-induced neutrophils from healthy controls

**Figure 6:**
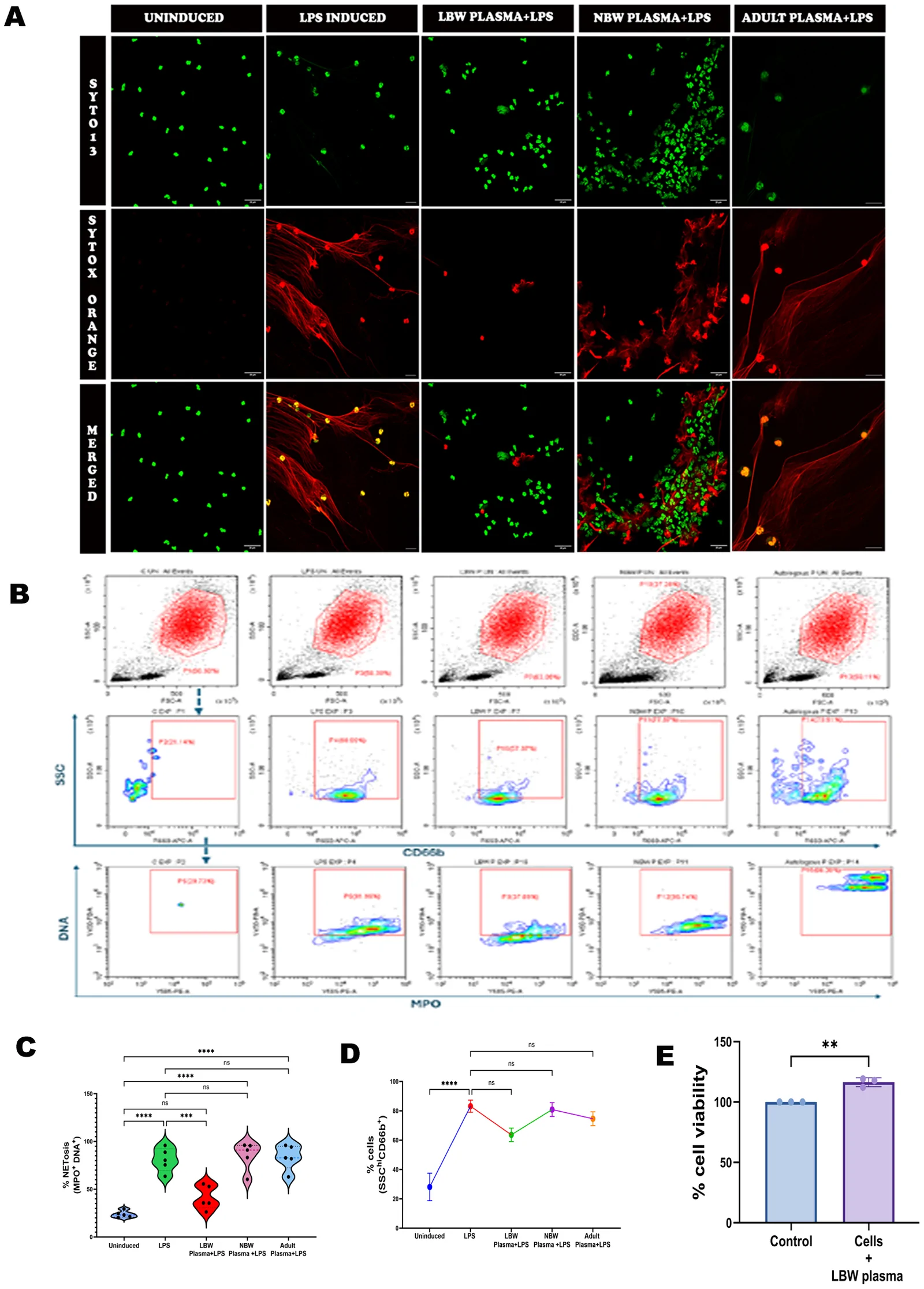
Evaluation of NETosis modulation by LP in healthy adult neutrophils. Representative data showing NET formation in LPS-stimulated neutrophils from healthy adults following incubation with LP. **(A)** Confocal microscopy images depict NET formation assessed by extracellular DNA staining (SYTOX Orange) and a cell-permeant intracellular DNA-binding dye (Syto13) (63× magnification; scale bar = 20 µm; n = 3). **(B)** Contour plots from flow cytometry data showing percentage of NET-forming neutrophils (n = 5) incubated with different LBW plasma samples. **(C)** Violin plot indicate proportion of MPO–DNA-positive neutrophils (n = 5). **(D)** Quantification of neutrophil activation based on CD66b expression. **(E)** Quantification of neutrophil viability (n = 3). All data are presented as mean ± SD. Statistical analysis was performed using one-way ANOVA **(C, D)** or Student’s t-test **(E)**. Significance is indicated as: non-significant (ns), p > 0.05, *p < 0.05, **p < 0.01, ***p <0.001, and ****p < 0.0001.

Extending our findings from SLE neutrophils, we next assessed the effect of LP on NET formation in neutrophils from healthy adults to distinguish whether the observed NET-inhibitory effects are intrinsic to neutrophil biology or driven by the inflammatory milieu present in SLE. High-resolution confocal microscopy revealed a significant reduction in extracellular DNA web formation in neutrophils from healthy controls upon treatment with LP (**Figure 6A**). In contrast, plasma from NBW newborns and autologous adult plasma failed to inhibit NET formation under similar conditions. For comparison, extracellular DNA staining using SYTOX Orange, together with intracellular DNA labeling by SYTO13, confirmed NET formation in LPS-stimulated neutrophils from healthy controls.

Myeloperoxidase (MPO), a key effector enzyme and hallmark of NETosis, forms extracellular MPO–DNA complexes that can contribute to inflammatory pathology if not adequately cleared. Consistent with the imaging data, LP significantly reduced both the proportion of netting neutrophils (**Figure 6B**) and the number of MPO-DNA-positive cells (**Figure 6C**) under LPS-stimulated conditions compared to NBW and autologous adult plasma. Furthermore, LP decreased neutrophil activation, as evidenced by reduced CD66b expression (**Figure 6D**).

To exclude potential cytotoxic effects, neutrophil viability was assessed using an MTT assay. LP treatment resulted in a significant increase in cell viability (**Figure 6E**), indicating that the observed inhibition of NETosis was not due to cytotoxicity but rather reflects a specific inhibitory effect.

### 3.2 Preclinical evaluation of LP in the PIL mice model

#### 3.2.1 PIL model development and its evaluation

Pristane administration in BALB/c mice led to the development of lupus-like disease within one month, as evidenced by the emergence of characteristic clinical manifestations. Compared to unimmunized controls, PIL mice displayed several clinical features that mirrored those of human SLE, confirming the successful establishment of the PIL model for subsequent evaluation.

##### 3.2.1.1 Autoantibody production

**Figure 7:**
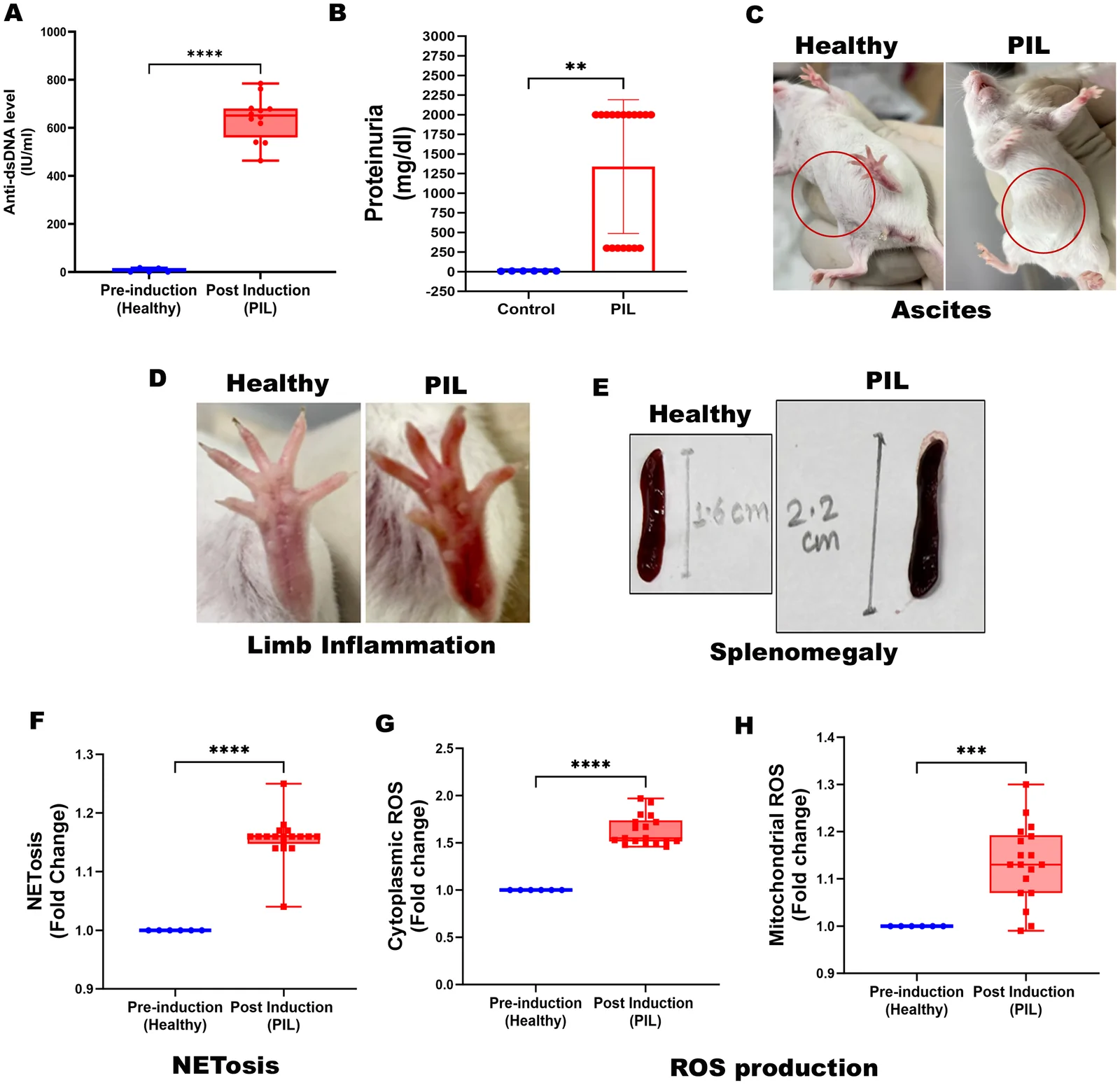
Clinical validation of PIL mouse model development. Representative data showing **(A)** serum anti-dsDNA antibody levels and **(B)** proteinuria (p=0.0009; n=6 per group) after one month of pristane injection. Additional physiological manifestations included **(C)** ascites development, **(D)** limb inflammation, and **(E)** splenomegaly. Quantification of **(F)** NETosis, **(G)** cytoplasmic ROS, and **(H)** mitochondrial ROS in PIL mice. Data are presented as mean ± SD (n = 18). Statistical analysis was performed using Student’s t-test, with significance indicated as: non-significant (ns), p > 0.05, *p < 0.05, **p < 0.01, ***p <0.001, and ****p < 0.0001.

PIL mice exhibited a significant increase in serum anti-dsDNA autoantibody levels compared to pretreatment baseline (**Figure 7A**), indicating the development of a hallmark feature of lupus.

##### 3.2.1.2 Proteinuria

A marked increase in urinary protein excretion was observed in PIL mice (**Figure 7B**), consistent with renal involvement and disease progression.

##### 3.2.1.3 Clinical and physiological manifestations

Additional phenotypic parameters, including ascites development (**Figure 7C**), limb inflammation (**Figure 7D**), and organomegaly (**Figure 7E**), were observed. Collectively, these findings confirmed the successful establishment of the lupus-like disease model and recapitulation of clinical features of SLE.

##### 3.2.1.4 NETosis

Given the central role of aberrant NETosis in lupus pathogenesis, neutrophil function was assessed in the PIL model. PIL mice showed significantly elevated NETosis after four weeks compared to baseline (**Figure 7F**), consistent with observations in patients with SLE and our *ex vivo* findings.

##### 3.2.1.5 ROS production

In parallel, ROS production was significantly increased in neutrophils from PIL mice (**Figures 7G, H**), highlighting enhanced oxidative stress. This elevated ROS generation supports the role of oxidative pathways in disease progression and further validates the PIL model for studying NETosis-targeted interventions.

Following successful confirmation of the lupus model, the PIL mice were randomized into three experimental groups (n = 6 per group) to evaluate the therapeutic efficacy of CB plasma. Group 1 served as the untreated lupus control and received normal saline, Group 2 received LBW newborns’ CB plasma, and Group 3 received NBW newborns’ CB plasma. Unimmunized mice (n = 6) were maintained as healthy controls to provide baseline comparator values across all measured endpoints. This experimental design enabled a systematic evaluation of LP in modulating NETosis-associated immunopathology compared to both untreated and NBW newborns’ CB plasma-treated cohorts.

#### 3.2.2 LP reduced NETosis, associated protein markers and ROS production in the blood-derived neutrophils of PIL model

**Figure 8:**
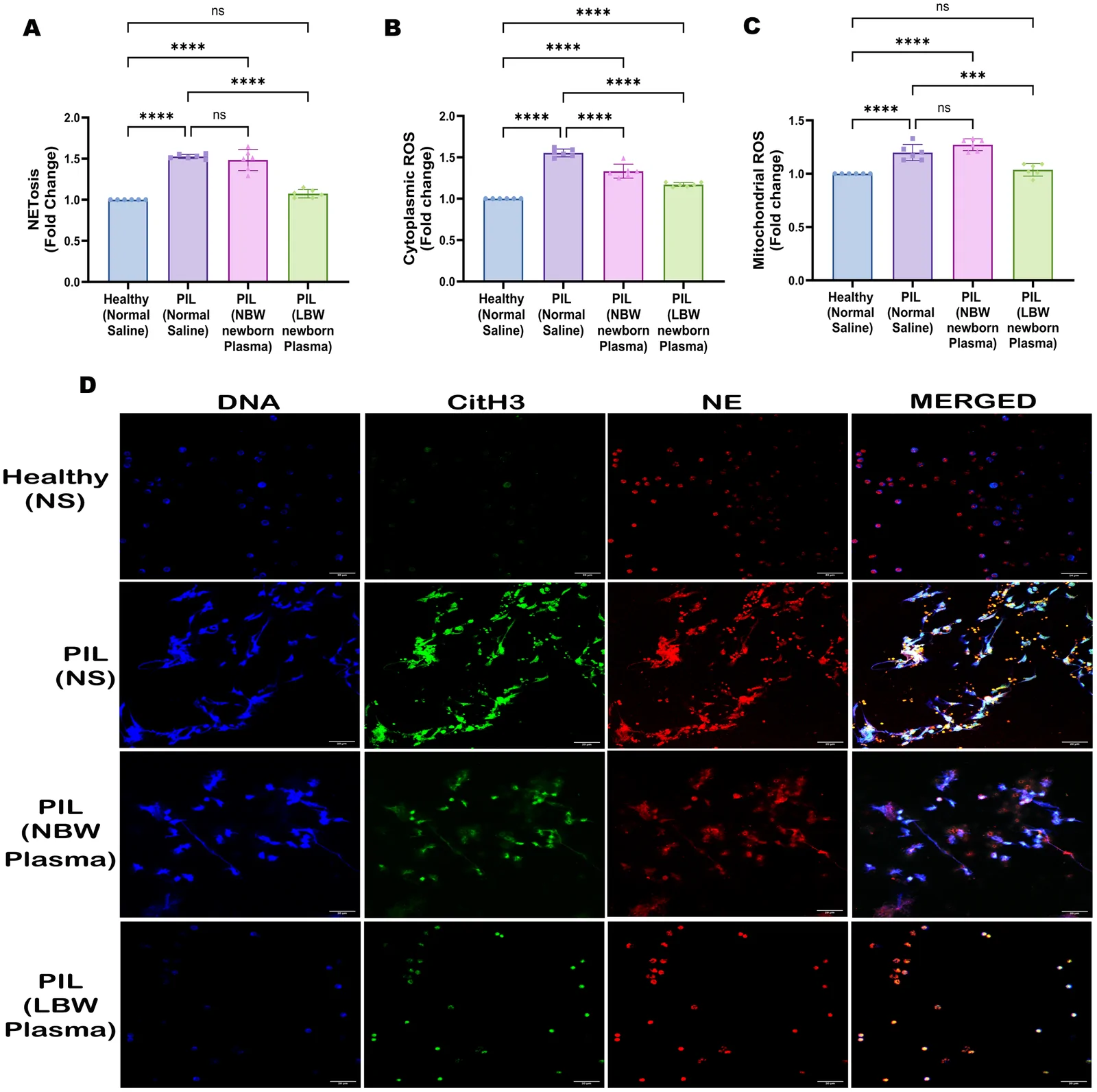
Immunomodulation of NETosis, ROS, and NETosis markers in PIL mice following LP administration. Representative data showing the quantification of **(A)** NETosis, **(B)** cytoplasmic ROS, **(C)** mitochondrial ROS, and **(D)** NETosis markers (CitH3 and NE) in the neutrophils of the PIL mice following three weeks of plasma administration. Bar graphs and confocal microscopy images illustrate mean data from five mice per group. Confocal microscopy images show expression of NETosis markers in neutrophils from PIL mice receiving LBW plasma, with netting neutrophils confirmed by co-localization of NE (red), CitH3 (green), and DNA **(blue)** (63× objective; scale bar = 20 µm). Data are presented as mean ± SD (n = 5). Statistical analysis was performed using one-way ANOVA, with significance indicated as: non-significant (ns), p > 0.05, *p < 0.05, **p < 0.01, ***p <0.001 and ****p < 0.0001.

To validate the *ex vivo* findings on the NETosis-inhibitory role of LP in neutrophils from SLE patients, pooled LBW or NBW newborns’ CB plasma was injected into their respective PIL mouse groups. Untreated PIL and healthy mice were administered with normal saline (NS). Following three weeks of plasma administration, a quantitative assessment of neutrophil culture supernatants demonstrated a30.93% reduction in NETosis in PIL mice treated with LP (**Figure 8A**) compared to untreated PIL mice that received NS, indicating a specific NETosis-inhibitory activity of LBW plasma.

Cytoplasmic (**Figure 8B**) and mitochondrial ROS (**Figure 8C**) levels were examined to assess oxidative stress. Consistent with the NETosis data, administration of LP resulted in a 15.34% and 24.28% reduction in mitochondrial and cytoplasmic ROS levels, respectively, compared to PIL mice that received NS. These findings suggest that LP attenuates oxidative stress implicated in lupus pathogenesis.

To further corroborate these findings, qualitative analyses were performed using confocal microscopy. NET formation and associated protein markers were examined, revealing attenuation of NET structures in neutrophils from LP-administered PIL mice (**Figure 8D**). Notably, the expression of canonical NETosis-associated proteins, including CitH3 and NE, was markedly diminished in this group compared to untreated and NBW newborns’ CB plasma-administered mice.

#### 3.2.3 LP suppressed NETosis in the heart, liver, and kidneys of PIL mice

The evaluation of extracellular DNA traps *in vivo* is inherently challenging because it requires specialized imaging strategies beyond simple DNA quantification. To address this challenge, we employed dual-contrast labeling with SYTOX Orange and myeloperoxidase (MPO) to quantify MPO–DNA complexes within the livers, kidneys, and hearts of PIL mice. The tissue-specific distribution of these NETosis markers was assessed using the Vevo LAZR-X *in vivo* imaging system (Fujifilm VisualSonics).

##### 3.2.3.1 Heart

**Figure 9:**
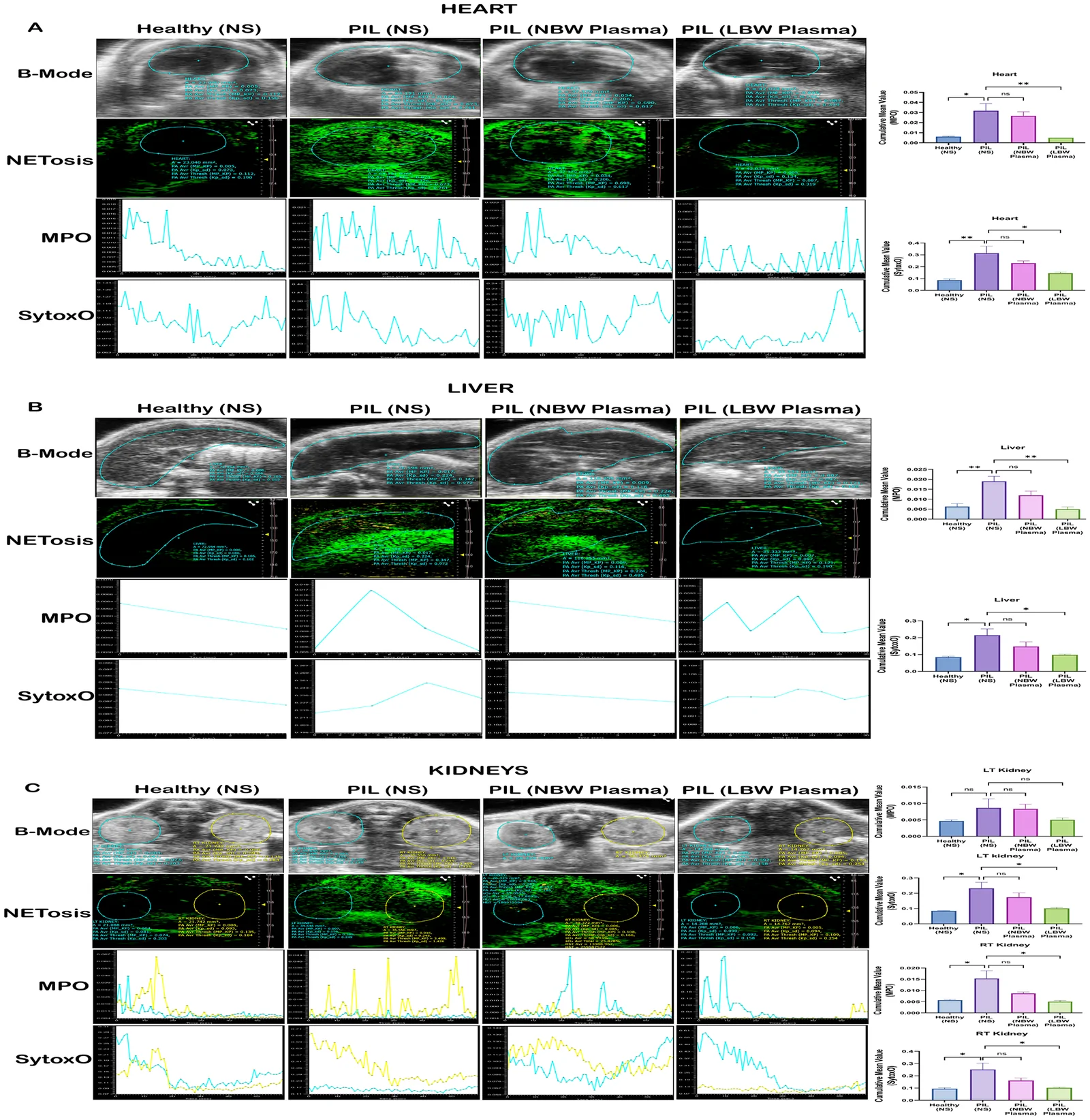
Modulation of systemic NET formation by LP in vital organs of PIL mice. Representative data showing the signal intensities of SYTOX Orange (extracellular DNA) and MPO in the **(A)** heart, **(B)** liver, and **(C)** kidneys following three weeks of plasma administration. Phantom imaging with dual contrast agents (SYTOX Orange and MPO) was used to assess NETosis *in vivo*. Line graphs show the distribution of SYTOX Orange and MPO across vital organs. Confined blue and yellow indicate the regions of interest (ROI) used for image quantification. The bar graphs show cumulative mean values of MPO and SYTOX Orange in the heart, liver, and kidneys (n = 3). Data are presented as mean ± SD. Statistical analysis was performed using one-way ANOVA, with significance indicated as: non-significant (ns), p > 0.05, *p < 0.05, **p < 0.01, ***p <0.001 and ****p < 0.0001.

The tissue-specific accumulation of SYTOX Orange and MPO was examined in the hearts of PIL mice. Using the brightness mode (B-mode), the region of interest (ROI) was manually defined, and NETosis was quantified. Our analyses revealed accumulation of SYTOX Orange and MPO across the heart of PIL mice that received normal saline (NS) compared to healthy controls, confirming enhanced *in vivo* NETosis. As shown in **Figure 9A**, PIL mice administered with LP demonstrated a significant reduction in both extracellular DNA (SYTOX Orange signal) and MPO accumulation in the heart.

##### 3.2.3.2 Liver

The NET burden was further evaluated in the livers of PIL mice three weeks after plasma administration. We observed a reduced signal intensity of both SYTOX Orange and MPO in PIL mice injected with LP compared with PIL mice that received normal saline. In contrast, NBW newborns’ CB plasma administration failed to reduce *in vivo* NETosis in the livers of PIL mice (**Figure 9B**).

##### 3.2.3.3 Kidneys

Similar to the heart and liver, live NETosis was quantified in the kidneys of PIL mice. Using grayscale ultrasound images (B-mode imaging), the ROI was defined to locate the left and right kidneys in the abdominal area of the mice. PA imaging showed a reduction in the accumulation of extracellular DNA (stained with SYTOX Orange) and MPO in the left and right kidneys of lupus mice administered with LP compared to PIL mice that received normal saline (**Figure 9C**).

#### 3.2.4 NETosis-modulating effects of LP on NET-driven inflammation and organ hypoxia in the PIL mice

**Figure 10:**
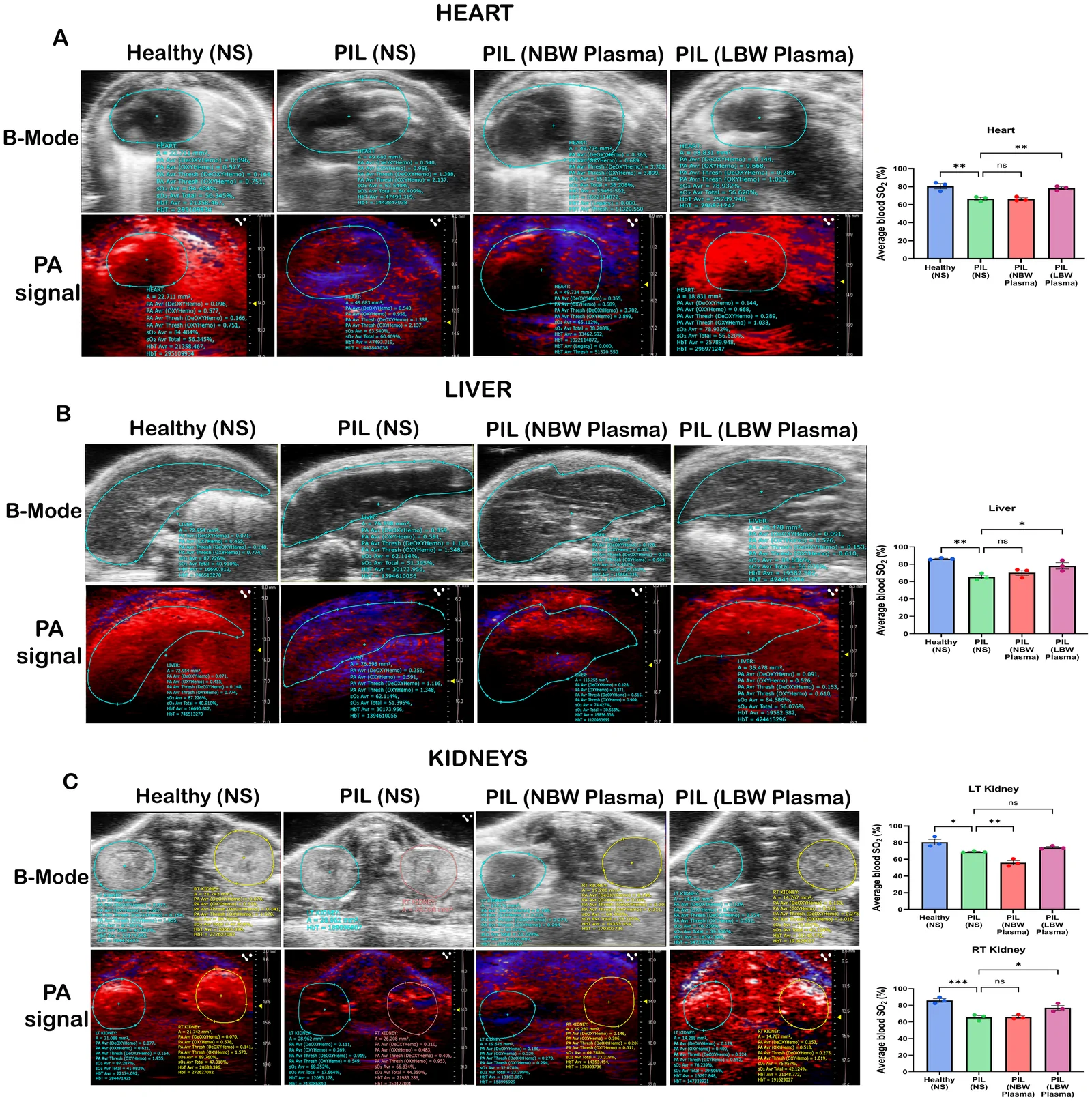
Assessment of organ oxygenation dynamics by dual-mode photoacoustic (PA) imaging in PIL mice. Representative PA images show the distribution of oxygenated (HbO_2_) and deoxygenated hemoglobin (Hb) in the **(A)** heart, **(B)** liver, and **(C)** kidneys, following treatment with LP. Highlighted blue and yellow regions denote the regions of interest (ROI) used for quantitative analysis. Bar graphs represent cumulative mean PA signal intensities of HbO_2_ and Hb in each organ (n = 3), reflecting changes in tissue oxygenation following LBW plasma administration. In the PA images, red signals correspond to HbO_2_, whereas blue signals indicate Hb. Data are presented as mean ± SD. Statistical analysis was performed using one-way ANOVA, with significance indicated as: ns (p > 0.05), *p < 0.05, **p < 0.01, ***p < 0.001, and ****p < 0.0001.

Functional PA imaging was performed to assess tissue oxygenation, vascular physiology, and pathological alterations, including inflammation, hypoxia, and ischemia. Ultrasound-guided PA images in oxy–deoxy mode were acquired. In this mode, oxygenated hemoglobin (HbO_2_) is represented in red, indicating well-oxygenated tissue, whereas deoxygenated hemoglobin (Hb) appears in blue, reflecting hypoxic or poorly perfused regions. Notably, treatment with LBW newborns’ cord blood (CB) plasma resulted in a marked increase in red signal intensity with a corresponding reduction in blue regions across the heart, liver, and kidneys, indicating improved tissue oxygenation and reduced hypoxia (**Figures 10A–C**). This was accompanied by attenuation of NETosis-associated inflammation and preservation of tissue integrity. In contrast, NBW newborns’ CB plasma failed to produce comparable changes in oxygenation or inflammatory status. Collectively, these findings demonstrate the inhibitory effects of LP in mitigating NETosis *in vivo* and highlight its potential in the modulation of NETosis in SLE.

#### 3.2.5 LP ameliorates lupus severity and restores organ integrity

**Figure 11:**
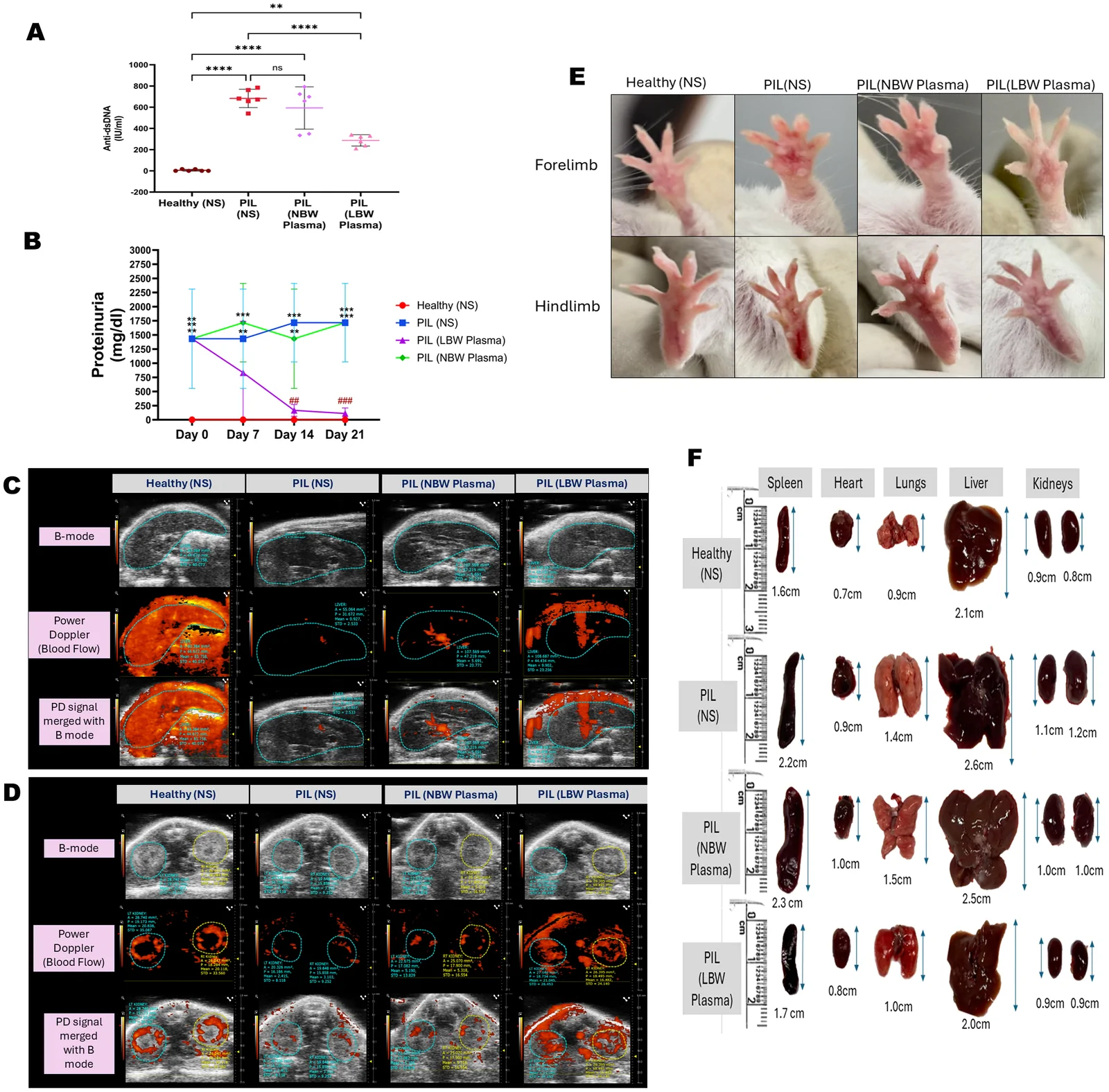
NETosis-modulating effects of LP on NETosis-associated inflammation, lupus pathology, and organ damage. Representative data showing **(A)** serum anti-dsDNA autoantibody levels and **(B)** proteinuria in PIL mice following treatment with LP. Panels **(C)** and **(D)** represent photoacoustic images displaying a B-mode ultrasound image overlaid on a Power Doppler image of the liver **(C)** and kidneys **(D)**, respectively. Panel **(E)** represents limb inflammation, and panel **(F)** represents the size of the affected organs in PIL mice following treatment with LP. Data are representative of three independent experiments.

Following three weeks of plasma administration, mice treated with LP exhibited a 57.9% reduction in serum anti-dsDNA autoantibody levels (**Figure 11A**), a key serological indicator of lupus activity. By day 21, these mice demonstrated a 93.59% (p=0.0009; n=6 per group) decrease in proteinuria compared with untreated PIL mice (**Figure 11B**). Notably, non-invasive *in vivo* assessment of vascular function using photoacoustic imaging (B-mode and PA-mode) revealed enhanced vascularity in the liver and kidneys (**Figures 11C, D**) of PIL mice treated with LP compared to untreated lupus controls and those that received NBW newborns’ CB plasma, suggesting a beneficial impact on the preservation of vascular integrity and perfusion.

In lupus, the pathological burden is largely orchestrated by dysregulated NETosis, which not only exposes nuclear antigens that promote anti-dsDNA autoantibody production but also sustains aberrant activation of innate immune sensors, dendritic cell maturation, and autoreactive T- and B-cell responses (18, 19). Consistent with its NETosis-inhibitory activity, LBW plasma treatment was associated with attenuation of lupus-associated pathology. Specifically, LBW plasma administration resulted in reduced systemic and tissue-level inflammation, as evidenced by diminished limb swelling (**Figure 11E**), improved vascular function, and restoration of morphological integrity in the vital organs of lupus mice (**Figure 11F**). While reduced NET burden may contribute to these effects, the present study did not directly evaluate the immunological mechanisms linking NETosis inhibition to decreased autoantibody production or disease amelioration.

## 4 Discussion

SLE is a complex autoimmune disorder characterized by a breakdown of immune tolerance, persistent inflammation, and progressive multiorgan damage. Increasing evidence implicates NETosis as an important contributor to disease pathogenesis. Consequently, modulation of NETosis has emerged as an area of increasing interest in the development of strategies aimed at limiting NET-associated pathology. Our previous work demonstrated that dexamethasone- and IFN-γ–primed mesenchymal stem cell–conditioned media effectively suppress NETosis in SLE neutrophils, providing proof-of-concept for targeting NET-mediated pathology (20). In the present study, we show that LP possesses NETosis-inhibitory activity and is able to suppress pathological NET formation and inflammatory responses, both *ex vivo* in neutrophils from patients with SLE and *in vivo* in a PIL mouse model.

A key observation of this study was the significant attenuation of extracellular DNA release along with diminished expression of NET-associated markers, including CitH3 and NE, in patients’ neutrophils when exposed to LBW newborns’ CB plasma. Excessive NETosis in SLE is well documented and is thought to contribute to the generation of autoantigens and immunostimulatory complexes that drive the production of autoantibodies and perpetuate inflammatory circuits. The ability of LBW plasma to attenuate NET formation under both spontaneous and LPS-stimulated conditions suggests its NETosis-inhibitory activity capable of modulating neutrophil responses. Importantly, the mechanisms responsible for impaired NETosis in tLBW newborns remain incompletely understood. Previous studies from our group have demonstrated intrinsic alterations in neutrophil signaling pathways and NETosis-associated machinery in this population. However, the contribution of soluble circulating factors has remained unclear. The present findings provide evidence consistent with the existence of plasma-derived NET-regulatory activity and support further investigation into its molecular basis.

An informative aspect of the study was the differential response observed between LBW and NBW CB plasma. While LBW plasma consistently reduced NET formation and associated inflammatory parameters, NBW plasma failed to exert comparable effects. This observation suggests that the inhibitory activity identified in the present study is unlikely to represent a universal property of neonatal cord blood plasma. Several explanations may account for this difference. It is possible that LBW plasma contains higher concentrations of putative NET-inhibitory factors, distinct molecular variants, or combinations of factors not present in NBW plasma. Alternatively, the observed activity may reflect developmental adaptations associated with fetal growth restriction or altered intrauterine environments. Although the present study was not designed to distinguish among these possibilities, the contrasting responses observed between LBW and NBW plasma highlight the importance of further molecular characterization.

The observed suppression of NETosis by LP is closely aligned to its ability to reduce ROS production, as NET formation is tightly coupled to oxidative pathways, including NADPH oxidase-driven cytoplasmic ROS and mitochondrial ROS generation. In SLE, excessive ROS production not only drives NET formation but also contributes to endothelial tissue damage and amplification of the inflammatory response (21). The observed attenuation of mitochondrial and cytoplasmic ROS generation in LBW plasma-treated SLE neutrophils suggests that LP may influence the upstream regulatory nodes required for the functional NET formation. This concurrent reduction in ROS production and NET formation may reflect broader modulation of neutrophil activation pathways rather than inhibition of a single downstream process. Further, since selective ROS pathway inhibition was not performed, the present study does not establish whether ROS suppression is necessary or sufficient for the observed NETosis-inhibitory effects. The concomitant reduction in NET formation underscores the crucial role of oxidative stress in NET-driven autoimmune pathology and suggests that LP may influence redox-dependent pathways involved in immune dysfunction.

In addition to restricting NET release, LP lowered the proportion of neutrophils undergoing NETosis, as reflected by reduced MPO–DNA complex formation. Notably, this effect was evident in both spontaneous and LPS-induced states, indicating consistent inhibitory activity across both spontaneous and stimulated conditions. Further, the reduction in CD66b expression observed following LBW plasma treatment may indicate effects beyond NET formation alone and warrants further investigation. Importantly, the absence of cytotoxicity, together with preserved or enhanced neutrophil viability, underscores that LBW plasma selectively restrains pathological activation without impairing overall neutrophil function.

The biological relevance of these observations was further explored using the PIL model. PIL mice exhibited an elevation in serum anti-dsDNA autoantibody levels, increased proteinuria, ROS production and NETosis, confirming successful development of lupus-like disease. Administration of LP resulted in reduced NETosis, oxidative stress, and canonical NET-associated markers, including CitH3 and NE in the neutrophils of the PIL mice. Reduced accumulation of MPO-DNA complexes was also observed in the heart, liver, and kidneys of PIL mice. Notably, these changes were accompanied by reductions in proteinuria and anti-dsDNA autoantibody levels, along with preserving vascular integrity, and improving tissue oxygenation. Although NET-derived nuclear components have been implicated in autoreactive immune responses and autoantibody generation in SLE, the present study did not directly assess adaptive immune mechanisms, including B-cell activation, T-cell responses, plasmablast differentiation, interferon signaling, or complement activation. Consequently, the observed reduction in autoantibody levels may not be attributed solely to reduced NET burden. Additional immunological pathways may also contribute to the beneficial effects observed following LBW plasma administration.

The present findings should also be interpreted in the context of previous reports describing neonatal NET-inhibitory factors (nNIFs), endogenous cord blood-derived molecules that transiently suppress NET formation during early life and protect against excessive inflammatory responses (17). Consistent with these observations, LP significantly reduced NET formation in SLE neutrophils and NETosis-associated pathologies in the PIL model, suggesting the presence of soluble NET-regulatory activity. Furthermore, nNIF administration has been shown to inhibit NET formation and improve outcomes in experimental polymicrobial sepsis, supporting the broader therapeutic potential of neonatal NET-regulatory mechanisms (22). However, the present study did not isolate, identify, or quantify specific inhibitory molecules within LBW CB plasma. Therefore, while the observed activity is analogous to that reported for nNIFs, the distinct factors unique to LBW newborns that mediate such inhibitory activity cannot be defined. Future studies involving biochemical fractionation, proteomic characterization, and functional validation will be required to identify the molecular factors responsible for the observed activity.

Collectively, our findings demonstrate that CB plasma derived from term LBW newborns possesses NETosis-inhibitory activity capable of attenuating NETosis and associated inflammatory responses in the PIL mouse and *ex vivo* SLE neutrophil system. While the molecular basis of this activity remains undefined, the results support the concept that naturally occurring as-yet-uncharacterized plasma-derived regulatory mechanisms may contribute to the restrained NETotic phenotype previously observed in LBW newborns. Further mechanistic studies will be necessary to identify the responsible factors, define their mode of action, and determine their broader biological significance in NET-driven inflammatory diseases.

## 5 Study limitations

Several limitations of the present study should be acknowledged. First, NETosis was assessed using LPS as the sole inducer. Because NET formation can occur through multiple mechanistically distinct pathways depending on the stimulus, the effects of LP on NETosis induced by alternative stimuli, including PMA, calcium ionophores, immune complexes, or lupus-associated autoantibodies, remain unknown and need to be investigated further. Second, standardized disease activity measures such as SLEDAI or BILAG were not available for all recruited patients and therefore could not be incorporated into the analysis. As disease activity may influence neutrophil activation and NETosis, future studies should include comprehensive clinical activity assessment. Third, the patient cohort predominantly comprised individuals with glomerulonephritis and/or pericarditis recruited from a tertiary-care referral center, which may limit the generalizability of the findings across the broader spectrum of SLE phenotypes. Fourth, the study did not employ selective ROS inhibitors or rescue experiments to determine whether ROS suppression is necessary and/or sufficient for the observed NETosis-inhibitory effects. Future studies employing targeted pharmacological or genetic approaches will be required to define the mechanistic relationship between ROS modulation and NET inhibition. Fifth, the molecular identity of the NETosis-modulating activity present in LBW cord blood plasma was not determined, precluding definitive conclusions regarding the responsible mediators and their mechanisms of action. Sixth, although the PIL mouse reproduces several important features of human SLE, it does not fully capture the genetic, clinical and immunological heterogeneity of the human disease. Finally, the present study did not evaluate the biodistribution, tissue targeting, persistence, or pharmacokinetic properties of plasma-derived inhibitory factors following systemic administration. Consequently, the mechanisms through which these factors exert their *in vivo* effects remain to be established.

## Data Availability

The original contributions presented in the study are included in the article/**Supplementary Material**. Further inquiries can be directed to the corresponding author.
