# Supplementary material for: NETosis inhibitory activity of LBW newborns’ cord blood plasma reverses lupus-associated immunopathology
Source: Front Immunol. 2026 Aug 18;17:1863546. doi: 10.3389/fimmu.2026.1863546 (PMC13530011; doi:10.3389/fimmu.2026.1863546)
Supplement: Supplementary file 1 [file Table1.docx]

| S. No.  **Table S1: Details of the newborns recruited for the study** | Newborn with ID | Gender | Birth weight  (g) | Gestation age | Type of delivery | APGAR Score |
| --- | --- | --- | --- | --- | --- | --- |
| 1. | LBW50 | M | 2320 | 37+1 | NORMAL | 8/10,9/10 |
| 2. | LBW51 | M | 2360 | 38+1 | NORMAL | 6/10,7/10 |
| 3. | LBW52 | F | 2240 | 39+0 | NORMAL | 8/10,9/10 |
| 4. | LBW53 | M | 2320 | 37+0 | NORMAL | 8/10,9/10 |
| 5. | LBW54 | F | 1650 | 38+4 | NORMAL | 8/10,9/10 |
| 6. | LBW55 | F | 2150 | 37+0 | NORMAL | 8/10,9/10 |
| 7. | LBW56 | F | 2180 | 39+3 | NORMAL | 8/10,9/10 |
| 8. | LBW57 | F | 2180 | 37+2 | NORMAL | 8/10,9/10 |
| 9. | LBW58 | F | 2010 | 39+2 | NORMAL | 8/10,9/10 |
| 10. | LBW59 | F | 1950 | 38+1 | NORMAL | 8/10,9/10 |
| 11. | LBW60 | F | 2025 | 39+0 | NORMAL | 8/10,9/10 |
| 12. | LBW61 | F | 2085 | 38+0 | LSCS | 8/10,9/10 |
| 13. | LBW62 | F | 2190 | 37+3 | NORMAL | 8/10,9/10 |
| 14. | LBW63 | F | 2135 | 37+6 | LSCS | 8/10,9/10 |
| 15. | LBW64 | F | 2060 | 39+0 | EMCS | 8/10,9/10 |
| 16. | LBW65 | F | 2225 | 39+2 | NORMAL | 8/10,9/10 |
| 17. | LBW66 | F | 2200 | 37+6 | LSCS | 8/10,9/10 |
| 18. | LBW67 | M | 2280 | 37+1 | NORMAL | 8/10,9/10 |
| 19. | LBW68 | F | 2260 | 39+4 | NORMAL | 8/10,9/10 |
| 20. | LBW69 | F | 2085 | 38+0 | LSCS | 8/10,9/10 |
| 21. | NBW78 | F | 3000 | 37+6 | NORMAL | 8/10,9/10 |
| 22. | NBW79 | F | 3100 | 38+3 | NORMAL | 8/10,9/10 |
| 23. | NBW80 | F | 3310 | 39+4 | NORMAL | 8/10,9/10 |
| 24. | NBW81 | F | 2953 | 38+0 | NORMAL | 8/10,9/10 |
| 25. | NBW84 | M | 3350 | 38+3 | NORMAL | 8/10,9/10 |
| 26. | NBW85 | F | 2915 | 37+3 | NORMAL | 8/10,9/10 |
| 27. | NBW86 | F | 3160 | 38+6 | NORMAL | 8/10,9/10 |
| 28. | NBW87 | F | 2710 | 41+4 | NORMAL | 8/10,9/10 |
| 29. | NBW88 | M | 3550 | 39+3 | NORMAL | 8/10,9/10 |
| 30. | NBW89 | F | 3510 | 36+2 | LSCS | 8/10,9/10 |
| 31. | NBW90 | F | 3080 | 39+0 | NORMAL | 8/10,9/10 |
| 32. | NBW91 | M | 2930 | 38+0 | NORMAL | 8/10,9/10 |
| 33. | NBW94 | M | 2950 | 39+4 | NORMAL | 8/10,9/10 |
| 34. | NBW95 | M | 3200 | 37+2 | NORMAL | 8/10,9/10 |
| 35. | NBW96 | F | 3645 | 39+1 | LSCS | 8/10,9/10 |
| 36. | NBW97 | M | 3390 | 39+2 | NORMAL | 8/10,9/10 |
| 37. | NBW98 | M | 3090 | 39+0 | NORMAL | 8/10,9/10 |
| 38. | NBW99 | F | 2870 | 41+0 | NORMAL | 8/10,9/10 |
| 39. | NBW100 | F | 3190 | 38+4 | NORMAL | 8/10,9/10 |
| 40. | NBW101 | F | 2920 | 38+0 | NORMAL | 8/10,9/10 |
