# Supplementary material for: NETosis inhibitory activity of LBW newborns’ cord blood plasma reverses lupus-associated immunopathology
Source: Front Immunol. 2026 Aug 18;17:1863546. doi: 10.3389/fimmu.2026.1863546 (PMC13530011; doi:10.3389/fimmu.2026.1863546)
Supplement: Supplementary file 2 [file Table2.docx]

| S. No.  **Table S2: Clinical profiles of patients with SLE** | Patient with ID | Age | Gender | Diagnosis | SLEDAI-2K score | Symptoms | dsDNA | ANA | Medications |
| --- | --- | --- | --- | --- | --- | --- | --- | --- | --- |
| 1 | S-150 | 19 | F | SLE | NA | Joint pain,  Malar rash, Photosensitivity  Hair loss | +ve | +ve | HCQ, Omnacortil,  Shelcal HD tab |
| 2 | S-151 | 28 | F | SLE with Lupus Nephritis, CNS Lupus, seizure disorder | NA | Glomerulonephritis | +ve | +ve | HCQ |
| 3 | S-152 | 19 | M | Lupus Nephritis | NA | Malar Rash, Hair loss | + ve | NA | HCQ |
| 4 | S-153 | 23 | F | SLE with Lupus Nephritis | 21 | Glomerulonephritis | +ve | NA | HCQ |
| 5 | S-154 | 22 | F | SLE with Lupus Nephritis (Proteinuria) with Post-Partum with flare | 15 | Glomerulonephritis | +ve | +ve | Omnacortil, HCQ, Shelcal |
| 6 | S-155 | 19 | F | SLE with Major organ | NA | Glomerulonephritis | +ve | +ve | Meropenem, Linezolid |
| 7 | S-156 | 24 | F | SLE (Diffuse cutaneous LE) | NA | Joint pain,  Malar rash, Hair loss, oral ulcers | +ve | +ve | HCQ, Solumedrol, PCM |
| 8 | S-157 | 40 | F | SLE+MCTD | NA | Joint pain, Hair loss, oral ulcers, Liver complications | +ve | +ve | HCQ, Shelcal, pantocid |
| 9 | S-158 | 26 | F | SLE with Lupus Nephritis | NA | Glomerulonephritis, Hair loss | +ve | +ve | HCQ, Shelcal, Omnacortil |
| 10 | S-159 | 18 | F | Lupus Nephritis, Recurrent loss of consciousness | NA | Glomerulonephritis, Discoid rash | +ve | +ve | HCQ, Solumedrol, Gluconate |
| 11 | S-160 | 40 | F | Lupus Nephritis | NA | Photosensitivity | +ve | +ve | HCQ, Tramadol, Emeset |
| 12 | S-161 | 30 | F | SLE with Lupus Nephritis | NA | Liver complications | +ve | NA | MMF, Omnacortil, Ostral |
| 13 | S-162 | 20 | F | SLE | NA | Discoid rash | +ve | +ve | HCQ, Thyrox |
| 14 | S-163 | 36 | M | SLE | NA | Joint pain, Photosensitivity, Hair loss, Oral ulcers, Discoid rash, Liver complications | +ve | +ve | HCQ, Omnacortil, |
| 15 | S-164 | 19 | F | SLE with acute cutaneous Lupus | NA | Malar rash, Photosensitivity, oral ulcers, Liver complications | +ve |  | HCQ |
| 16 | S-165 | 32 | F | SLE | NA | Joint pain, Malar rash, Hair loss, oral ulcers | +ve | +ve | HCQ, Shelcal |
| 17 | S-166 | 30 | F | SLE with oral ulcerations and alopecia | NA | Joint pain,  Malar rash, Hair loss, oral ulcers | +ve | +ve | HCQ, Omnacortil, Shelcal |
| 18 | S-167 | 21 | F | SLE | NA | Malar rash, Photosensitivity | +ve | +ve | HCQ, Augmentin |
| 19 | S-168 | 18 | F | SLE | 13 | Joint pain, Hair loss, oral ulcers, Discoid rash | +ve | +ve | HCQ, Indocap |
| 20 | S-169 | 23 | F | Lupus Nephritis | NA | Joint pain, Photosensitivity, Hair loss, oral ulcers, Liver complications | +ve | NA | Cyclophosphamide |
| 21 | S-170 | 19 | F | SLE with organ involvement, vasculitis, Lupus Nephritis | 30 | Joint pain, Hair loss | +ve | +ve | HCQ, Omnacortil, Shelcal |
| 22 | S-171 | 24 | F | SLE with Major organ, Lupus Nephritis | 18 | Glomerulonephritis, Hair loss, oral ulcers | +ve | +ve | HCQ, Thyrox, PCM |
| 23 | S-172 | 23 | F | SLE | 15 | Joint pain, Hair loss, malar rash, photosensitivity | +ve | +ve | HCQ, Omnacortil, Shelcal |
| 24 | S-173 | 25 | F | SLE with Lupus Nephritis | 22 | Joint pain, Glomerulonephritis | +ve | NA | HCQ, Shelcal, Bactrim |
| 25 | S-174 | 36 | F | SLE | NA | Joint pain, Photosensitivity, oral ulcers | +ve | +ve | HCQ, Shelcal, Bactrim |
| 26 | S-175 | 23 | F | SLE with organ involvement | NA | Glomerulonephritis, Hair loss, Discoid rash | +ve | +ve | Omnacortil, Shelcal, PCM |
| 27 | S-176 | 24 | F | SLE | NA | Joint pain, oral ulcers | +ve | +ve | HCQ, Emeset |
| 28 | S-177 | 24 | F | SLE | NA | Hair loss | +ve | NA | Folvite |
| 29 | S-178 | 30 | F | SLE with Lupus Nephritis | NA | Joint pain, oral ulcers | +ve | +ve | HCQ, Shelcal, Omnacortil |
| 30 | S-179 | 38 | F | SLE | NA | Joint pain, Liver complications | +ve | +ve | HCQ |
| 31 | S-180 | 25 | F | Lupus Nephritis | NA | Joint pain,  Malar rash, Hair loss | +ve | +ve | HCQ, Shelcal, Omnacortil |
| 32 | S-181 | 26 | F | Lupus Nephritis | >12 | Glomerulonephritis, Hair loss | +ve | NA | HCQ, Shelcal, Omnacortil |
| 33 | S-182 | 20 | F | SLE | NA | Malar rash, Hair loss, Discoid rash | +ve | +ve | HCQ |
| 34 | S-183 | 23 | F | SLE | NA | Malar rash, oral ulcers, joint pain, Hair loss | +ve | +ve | HCQ |
| 35 | S-184 | 26 | F | Lupus Nephritis | NA | Glomerulonephritis, Hair loss, Malar rash | +ve | NA | HCQ, Shelcal, Omnacortil |
